# An Edge-Deployable Method for Cow-Head Detection and Cross-Camera Association in Visible–Thermal Robotic Dairy Monitoring

**DOI:** 10.3390/ani16162528

**Published:** 2026-08-13

**Authors:** Chenxu Zhao, Fantao Kong, Zhiyong Zhang, Chenyang Zhang, Wei Sun, Shanshan Cao

**Affiliations:** 1 College of Computer and Information Engineering, Xinjiang Agricultural University, Urumqi 830052, China; 2 Agricultural Information Institute, Chinese Academy of Agricultural Sciences, Beijing 100081, China; 3 National Nanfan Research Institute (Sanya), Chinese Academy of Agricultural Sciences, Sanya 572024, China; 4 Institute of Agricultural Economics and Development, Chinese Academy of Agricultural Sciences, Beijing 100081, China

**Keywords:** precision livestock farming, dairy-cow monitoring, visible–thermal imaging, cow-head detection, heterogeneous-camera association, facial thermography, edge deployment

## Abstract

Facial surface temperature provides useful non-contact information for monitoring dairy-cow health, welfare, heat stress, and reproductive status. Infrared thermography can capture thermal information from facial regions such as the eyes, muzzle, nostrils, and ears; however, infrared images often contain weak texture and indistinct anatomical boundaries, which can hinder reliable cow-head localization during mobile robotic inspection. Visible-light images provide richer structural information but do not contain temperature data. This study developed YOLO11-AFE, a lightweight visible–thermal cow-head detection and heterogeneous-camera association method for quadruped inspection robots. The detector incorporates ADown for lightweight downsampling, C3k2_FE for adaptive feature enhancement, and SPPF_ECA for channel-aware multi-scale representation. An improved Hungarian matching algorithm was subsequently used to establish one-to-one correspondences between cow-head detections in synchronized visible-light and infrared pseudo-colour images. Across three independent runs, YOLO11-AFE achieved precision, mAP@0.5, and mAP@0.5:0.95 values of 97.59 ± 0.20%, 96.37 ± 0.24%, and 70.76 ± 0.51%, respectively, on the visible-light test subset, and 96.11 ± 0.24%, 99.07 ± 0.10%, and 91.50 ± 0.40%, respectively, on the infrared subset. The model required 2.14 million parameters and 5.27 GFLOPs, representing reductions of 17.37% and 18.17%, respectively, relative to YOLO11n. The association method achieved an overall accuracy of 98.11% across 371 ground-truth cow-head pairs in the combined validation and test evaluation. TensorRT FP16 deployment on the Jetson Orin NX achieved 36.71 FPS for the complete core processing pipeline. These results demonstrate that YOLO11-AFE provides an accurate and computationally efficient perception front end for future non-contact facial-temperature monitoring using mobile inspection robots.

## Simple Summary

In this study, we address the difficulties of non-contact facial temperature monitoring in dairy cows during robotic inspection by introducing a lightweight visible-light and thermal-infrared cow-head detection and association method. Thermal cameras can provide useful temperature information from facial regions such as the eyes, muzzle, nostrils, and ears, but thermal images often lack clear texture and anatomical details. Visible-light images contain clearer structural information, but they cannot provide temperature data. To overcome this limitation, the proposed method first detects cow heads in both visible-light and thermal images collected by a quadruped inspection robot. Subsequently, an improved matching algorithm is used to associate the same cow head between the two camera views. In tests conducted on synchronized visible-light and thermal image pairs from real dairy-farm scenes, the method achieved high detection accuracy, an overall association accuracy of 98.11% across 371 manually confirmed cow-head pairs, and real-time processing speed on an edge-computing platform. This research provides a practical front-end basis for future non-contact facial temperature monitoring and is meaningful for improving animal welfare and intelligent dairy-farm management.

## 1. Introduction

Continuous and low-disturbance monitoring is important for precision dairy-cow management. Variations in body temperature can provide useful information related to thermoregulation, heat stress, inflammatory responses, disease risk, and reproductive activity [1,2,3]. Infrared thermography offers a non-contact alternative to rectal thermometry and wearable sensors by capturing surface thermal radiation without restraining animals or attaching sensing devices [4,5]. Facial regions such as the eyes, muzzle, nostrils, ears, and horns have consequently been investigated as potential thermal indicators for dairy-cow health, welfare, and reproductive monitoring [6,7,8]. However, reliable non-contact automated body-temperature monitoring requires the relevant facial regions to be localized accurately and the resulting thermal information to be assigned to the correct animal. Cow-head localization and cross-camera target association are therefore essential front-end perception tasks for future robotic thermal monitoring.

Performing these tasks during mobile robot inspection remains challenging. Infrared pseudo-colour images provide thermal information but commonly contain weak texture, blurred anatomical boundaries, and limited structural details. These characteristics make reliable cow-head localization difficult, particularly in multi-cow scenes involving target overlap, fence occlusion, posture variation, and changing robot viewpoints. Robot motion may additionally introduce camera vibration and continuously varying observation angles, while the limited computational resources of an onboard edge platform constrain model size and processing complexity [9,10]. Therefore, relying only on a single infrared image stream may not provide sufficiently stable structural information for cow-head localization and target separation during routine robotic inspection.

Visible-light and infrared cameras provide complementary information. Visible-light images contain clearer contours, textures, and anatomical structures that support cow-head localization and separation, whereas infrared images retain the thermal information required for subsequent temperature analysis. However, independently detecting cow heads in the two modalities is insufficient. Each cow-head detection in the visible-light image must be associated with the corresponding detection in the synchronized infrared image. Incorrect association may assign thermal information to the wrong animal, especially when the two cameras differ in resolution, field of view, installation position, or observed target number. A deployable robotic monitoring system therefore requires both lightweight cow-head detection and target-level one-to-one association across heterogeneous camera views.

Vision-based livestock perception has been widely investigated for cattle detection, individual identification, behaviour recognition, tracking, and health monitoring. Existing studies have demonstrated the feasibility of deep learning for dairy-cow detection in barn environments, robot-assisted behaviour recognition, and automated livestock monitoring [11,12,13]. Computer vision and infrared thermography have also been applied to disease-related localization, while cow-head detection and tracking have supported multi-animal behaviour analysis [14,15]. Mar et al. developed a video-based cow detection and multi-object tracking method that associated cow regions across successive frames using centroid location, colour, texture, and CNN features [16]. Although this method demonstrates the value of combining spatial and appearance cues for cattle tracking, it addresses temporal association within a single visible-camera stream rather than instantaneous target-level association between synchronized visible-light and infrared camera views. Consequently, existing livestock-tracking methods cannot be directly applied to the heterogeneous-camera association task considered in this study.

Most existing visible–infrared studies have focused on image-level registration or multimodal fusion rather than target-level association. Image-registration methods generally estimate geometric correspondence through handcrafted or learned feature points, dense feature matching, cross-modal image generation, or global image transformations [17,18,19]. Studies of infrared–visible registration in complex scenes further indicate that weak texture, substantial cross-modal appearance differences, and geometric deformation can reduce registration stability [20,21,22]. Multimodal methods may also combine visible and thermal information at the pixel, feature, or decision level to improve object perception [23]. Depending on their architecture, these methods may require additional modality-specific feature extraction, spatial alignment, and cross-modal fusion operations, thereby increasing implementation complexity, memory consumption, and inference latency.

Pixel-level and feature-level fusion, in particular, generally require sufficiently consistent spatial and temporal correspondence between the two modalities. During mobile dairy inspection, differences in camera resolution, field of view, and installation position, together with robot motion, parallax, independently moving cows, fence occlusion, partial scene overlap, and unequal target numbers, make stable cross-modal alignment difficult [21,22]. Misalignment may cause unrelated structures or different animals to be combined, resulting in ghosting or unreliable cross-modal features [18]. Moreover, generating a fused image does not eliminate the need to retain the original radiometric infrared data for subsequent temperature extraction.

For these reasons, whole-image registration and dual-stream fusion were not used in this study. Instead, each visible-light image and its synchronized infrared pseudo-colour image were processed independently by the same shared lightweight detector. Target-level correspondence was then established between the resulting modality-specific cow-head boxes. This strategy preserves the native coordinate system and modality-specific information of each camera while reducing computational complexity and sensitivity to cross-modal misalignment.

**Figure 1:**
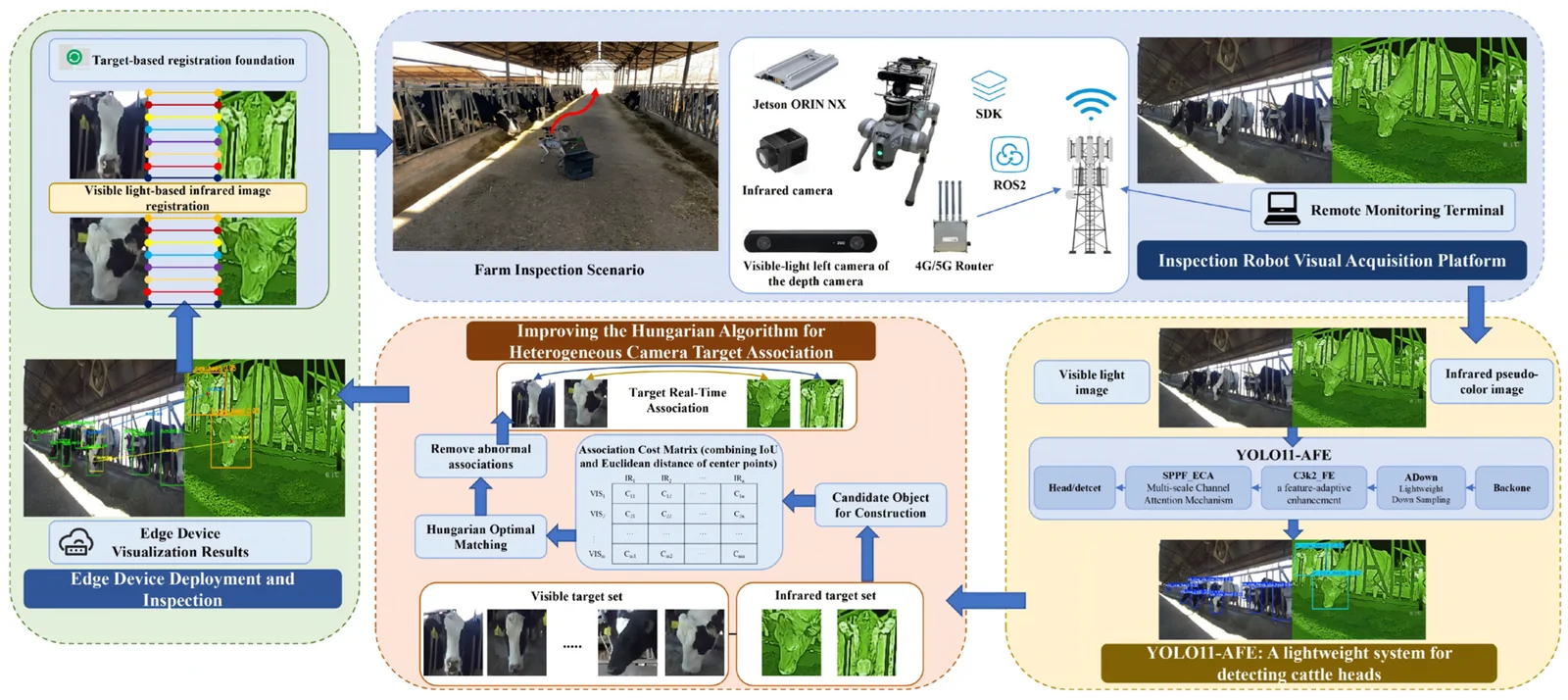
Overall workflow of visible–thermal cow-head detection and target association on the quadruped inspection robot.

Accordingly, this study proposes YOLO11-AFE, a lightweight method for visible–thermal cow-head detection and heterogeneous-camera target association on quadruped inspection robots (Figure 1). The proposed method focuses on two prerequisite perception tasks: detecting cow heads in visible-light and infrared pseudo-colour images and establishing partial one-to-one correspondence between synchronized cross-camera detections. It does not perform complete facial-temperature diagnosis. Instead, the resulting target-level correspondence provides an edge-deployable front-end basis for subsequent local registration, facial-region localization, radiometric temperature mapping, and non-contact temperature extraction from regions such as the eyes, muzzle, nostrils, and ears.

The main contributions of this study are as follows. First, a lightweight cow-head detector was developed based on YOLO11n. ADown was introduced to reduce downsampling computation, C3k2_FE was designed to enhance adaptive feature representation across visible-light and infrared pseudo-colour images, and SPPF_ECA was constructed by integrating efficient channel attention into SPPF. Second, a robotic dual-camera cow-head dataset was constructed under normal illumination, weak illumination, local shadows, strong light, viewpoint variation, fence occlusion, and single- and multi-cow conditions. Third, a heterogeneous-camera target-association strategy was developed to handle unequal target numbers and cross-modal positional differences by combining IoU and relative centre-distance cues within an improved Hungarian association algorithm. Fourth, the complete detection–association pipeline was deployed on a Unitree Go2 quadruped robot equipped with an NVIDIA Jetson Orin NX platform, and PyTorch and TensorRT experiments were conducted to verify its real-time edge-deployment feasibility.

## 2. Materials and Methods

### 2.1. Data Acquisition and Processing

**Figure 2:**
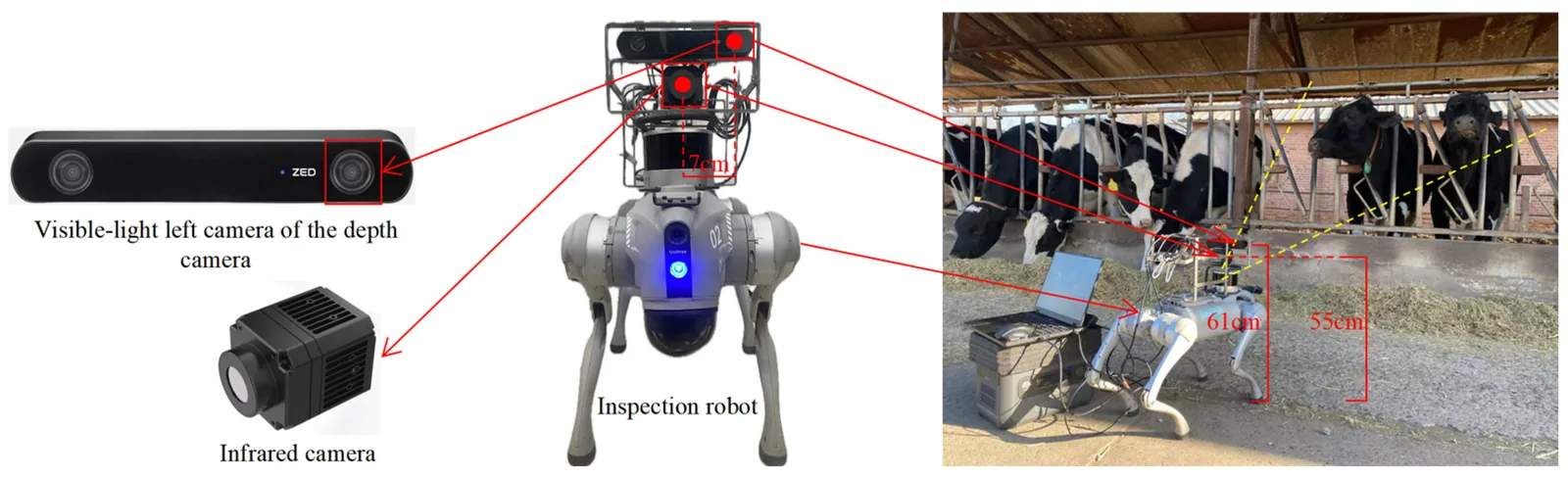
Visual perception system of the inspection robot.

Data were acquired at a dairy farm in the agricultural machinery test station of the Chinese Academy of Agricultural Mechanization Sciences Group Co., Ltd., Changping District, Beijing, China. Ten barns were included. To approximate the conditions faced by an inspection robot, the collection covered different cow postures, single- and multi-animal views, fence occlusion, normal lighting, weak illumination, local shadow, and strong-light interference. The sensing system was fixed on a Unitree Go2 quadruped robot (Unitree Robotics, Hangzhou, China), as shown in Figure 2, so the viewpoint was consistent with mobile inspection rather than a static camera setup.

For each acquisition cycle, visible images were captured by the left camera of a ZED2i depth camera (Stereolabs, San Francisco, CA, USA) at 1280 × 720 pixels, and infrared pseudo-colour images were captured by a Guide Sensmart IPN384 infrared camera (Wuhan Guide Sensmart Tech Co., Ltd., Wuhan, China) at 512 × 384 pixels. The SDK was extended so that both cameras were triggered at the same time. The interval between triggers was 2 s. Each trigger therefore generated one synchronized pair consisting of a visible-light image and the corresponding infrared pseudo-colour image. A total of 6973 raw image pairs were collected. Data acquisition was performed using a close-range patrol configuration to preserve sufficient cow-head and facial details for subsequent local visible–thermal registration, facial-region localization, and temperature extraction. Under this configuration, each frame generally covered a local area of the barn rather than the entire herd. In addition, the infrared camera provided more restricted effective scene coverage than the visible-light camera under the installed configuration. Consequently, most infrared pseudo-colour frames contained one or three clearly visible cow heads, whereas scenes containing more than five cow heads occurred relatively infrequently during routine robotic inspection.

Redundant synchronized image pairs were removed using the structural similarity index measure (SSIM) [24]. Because data acquisition was conducted separately in ten barns, SSIM-based filtering was also performed independently within each barn sequence. Temporally adjacent image pairs from different barns were not compared. This barn-specific procedure prevented differences in illumination, cow density, occlusion, and spatial layout among the barns from directly affecting the redundancy-removal decision.

Candidate SSIM thresholds of 0.65, 0.70, 0.75, 0.80, 0.85, and 0.90 were compared using the same 6973 raw synchronized image pairs. The corresponding numbers of retained image pairs were 767, 991, 1222, 1514, 2317, and 3855, respectively, as reported in Table S1. Because an adjacent image pair was treated as redundant when its SSIM value was equal to or greater than the specified threshold, lower thresholds resulted in more aggressive filtering, whereas higher thresholds retained more temporally repetitive observations.

The filtered sequences obtained under the candidate thresholds were reviewed barn by barn. A threshold of 0.75 provided the most appropriate balance between removing adjacent near-duplicate observations and retaining meaningful changes in cow position, posture, target arrangement, occlusion, and robot viewpoint. Thresholds of 0.65 and 0.70 produced more aggressive reduction, whereas thresholds of 0.80–0.90 retained progressively larger numbers of temporally repetitive observations. Therefore, 0.75 was selected for final dataset construction, resulting in 1222 retained synchronized image pairs.

The challenging acquisition conditions were primarily associated with the environments of particular barns rather than being randomly distributed across all image pairs. Among the ten barns, three were characterized by weak illumination, two by strong-light interference, two by normal natural illumination, and three by substantial local shadows. Dense cow distributions occurred in two weak-light barns, one strong-light barn, both naturally illuminated barns, and two shadow-affected barns. As summarized in Table S2, all ten barns and all seven barns containing dense cow distributions remained represented after filtering. The filtering procedure therefore reduced temporally repeated observations within each barn without eliminating any of the predefined barn-level illumination or cow-density categories.

An asymmetric annotation scheme was adopted because the two sensors differ in resolution, field of view, and imaging mechanism [25]. LabelImg was used to annotate the two modalities separately. The visible-light class was named vis_cow_head, and the infrared class was named ir_cow_head. For visible images, the annotation rule prioritized recall: all recognizable cow heads were labelled, including targets partly covered by fences, overlapping with other animals, or truncated at image borders. For infrared pseudo-colour images, the labels were designed to support subsequent facial-temperature monitoring. Only cow heads with clear anatomical structures, visible facial thermal cues, and no serious occlusion were retained, whereas irrelevant heat sources were excluded. The final annotations included 5683 cow-head instances in visible images and 1951 cow-head instances in infrared pseudo-colour images. The difference in the number of annotated instances therefore reflects both the modality-specific annotation criteria and the different effective scene coverage of the two cameras. Because the annotation criteria were modality-specific, a visible-light cow-head annotation did not necessarily have a corresponding infrared annotation in the same synchronized image pair. A cow head that was clearly recognizable in the visible-light image could be excluded from the infrared annotations when its anatomical structure or facial thermal cues were insufficient for subsequent temperature analysis. Such visible-only annotations were retained for visible-light detection training but were not treated as ground-truth cross-camera association pairs. A ground-truth association pair was defined only when valid cow-head annotations were present in both modalities and were manually confirmed to represent the same animal.

**Figure 3:**
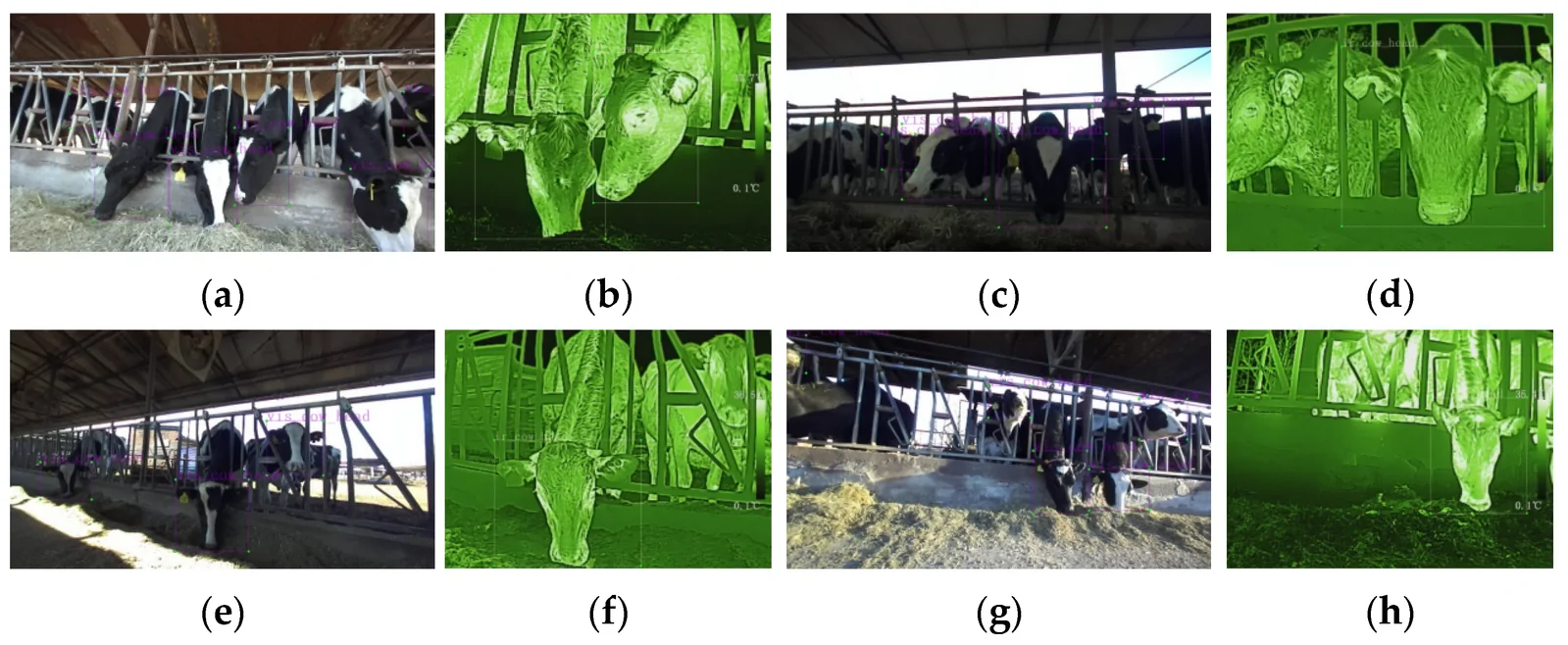
Example of visible and infrared pseudo-colour images under different illumination conditions. (**a**) Visible-light image under normal illumination; (**b**) Infrared pseudo-colour image under normal illumination; (**c**) Visible-light image under low-illumination conditions; (**d**) Infrared pseudo-colour image under low-illumination conditions; (**e**) Visible-light image under local shadow interference; (**f**) Infrared pseudo-colour image under local shadow interference; (**g**) Visible-light image under strong-light interference; (**h**) Infrared pseudo-colour image under strong-light interference.

Figure 3 shows representative labelled samples under several lighting conditions. The visible channel changes strongly with illumination, shadow, and reflection, whereas the infrared pseudo-colour channel is more stable to ambient visible light but provides much weaker texture information.

The data were divided into training, validation, and test subsets at about 8:1:1, while keeping samples independent for evaluation [26]. A total of 1956 images were used for training, 244 for validation, and 244 for testing in each modality. As shown in Table 1, the visible-light subset covered a wider range of scene densities, including crowded multi-cow scenes, whereas the infrared pseudo-colour subset was dominated by single-cow and two-cow scenes. This distribution reflects the practical close-range patrol configuration, the effective scene coverage of the installed cameras, and the modality-specific annotation criteria described above. Detection performance was therefore evaluated separately on the visible-light and infrared pseudo-colour test subsets. The modality-specific results were interpreted with consideration of their different instance counts and scene-density distributions rather than being treated as a direct like-for-like comparison between the two modalities.

The visible-light and infrared pseudo-colour images were combined into a single training dataset and used to train one shared YOLO11-AFE model jointly. The two modalities were neither introduced sequentially nor used to train two separate detectors. Each training sample contained an image from one modality, while the same network parameters were updated using samples from both modalities. The annotation categories were defined as vis_cow_head and ir_cow_head, enabling the shared detector to learn modality-specific cow-head representations within a unified model.

During inference, each visible-light image and its synchronized infrared pseudo-colour image were processed independently by the same detector. The resulting modality-specific bounding boxes were then passed to the heterogeneous-camera target-association module. Thus, the two modalities were jointly used during model training but were not fused at the image or feature level.

### 2.2. Construction of the Lightweight YOLO11-AFE Model

#### 2.2.1. Improvement of the YOLO11n Model

YOLO11n was chosen as the starting detector because the task requires fast cow-head perception from visible and infrared images in complex farm scenes. Its small parameter size and high inference speed make it practical for edge devices with limited resources [27]. Nevertheless, dual-light data introduce additional difficulties: visible images are disturbed by low light, shadows, and overexposure, whereas infrared pseudo-colour images often contain weak texture and simple colour patterns. The original YOLO11n therefore needs task-specific adaptation to represent both modalities effectively.

**Figure 4:**
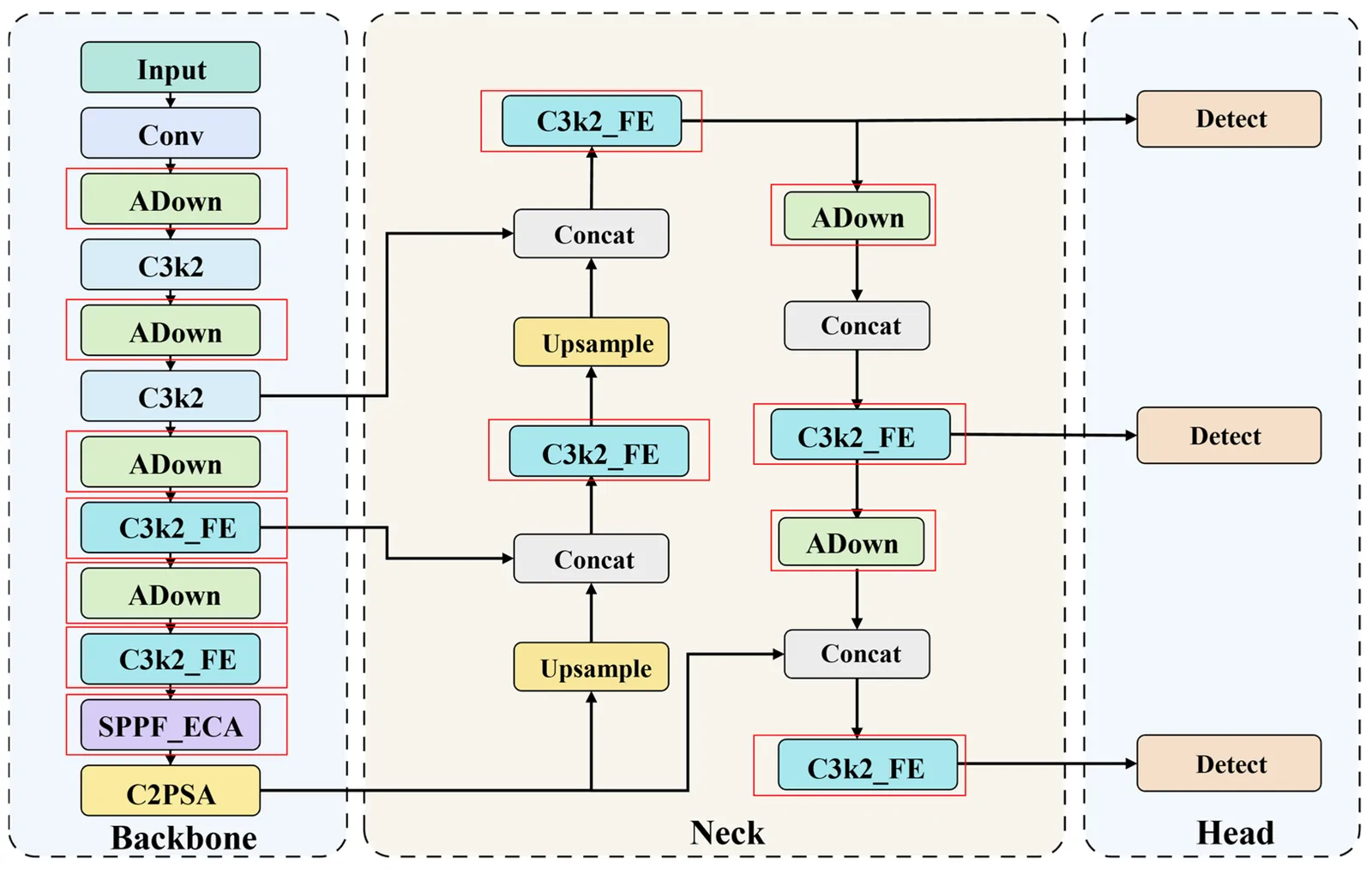
Overall architecture and inference process of the YOLO11-AFE lightweight cow-head detection model. The image first enters the backbone, where multi-scale semantic features are extracted (Step 1). ADown lowers downsampling computation and retains spatial cues, C3k2_FE adjusts cow-head features for different modalities, and SPPF_ECA enlarges the receptive field while highlighting informative channels. The neck then integrates and reconstructs features at multiple scales through ADown and C3k2_FE (Step 2). The detection head predicts cow-head boxes on several feature levels (Step 3), and decoding plus NMS produces the final detections (Step 4). The red boxes highlight the modules modified or introduced in YOLO11-AFE relative to the original YOLO11n architecture.

On this basis, the YOLO11-AFE detector was designed, where A, F, and E refer to ADown, FiLM Enhancement, and Efficient Channel Attention. The network in Figure 4 is composed of a backbone, neck, and detection head. In the backbone, some standard downsampling layers are replaced by ADown to reduce computation while retaining useful spatial responses. C3k2_FE is inserted into both the high-level backbone and the multi-scale neck to adapt features from visible and infrared inputs. ECA is further attached to SPPF, producing SPPF_ECA to strengthen multi-scale semantics and important channel responses. The Detect head from YOLO11n is retained to predict cow-head boxes at different scales. These changes aim to improve robustness to complex illumination and dual-light appearance variation while keeping the network compact.

The visible-light and infrared pseudo-colour images were combined into a unified training dataset and used to train one YOLO11-AFE model. Each training sample contained one image from either modality, and samples from both modalities were used to update the same set of network parameters. The two annotation categories, vis_cow_head and ir_cow_head, were learned within the same detector. During inference, each synchronized image pair was processed through two independent forward passes of the same network: one for the visible-light image and one for the infrared pseudo-colour image. The resulting modality-specific bounding boxes were then provided to the heterogeneous-camera target-association module.

YOLO11-AFE is a modality-shared single-stream detector rather than a dual-input multimodal fusion network. Each input sample contains either a visible-light image or an infrared pseudo-colour image, and one set of network weights is shared across both modalities. This design was selected according to the requirements of the subsequent association task, which requires cow-head detections to remain in the native coordinate system of each camera. A dual-stream fusion detector would generally require sufficiently aligned and synchronized image pairs or an additional registration module before cross-modal feature interaction. In the present robotic platform, the two cameras differ in resolution, field of view, and installation position, while robot motion, parallax, animal movement, occlusion, and unequal target numbers further reduce the reliability of pixel-level alignment. Modality-specific branches and cross-modal fusion modules would also increase memory consumption and inference complexity on the edge platform. Therefore, the shared single-stream design was adopted to balance cross-modal adaptability, independent modality processing, and real-time edge deployment.

#### 2.2.2. Introduction of the ADown Lightweight Downsampling Module

**Figure 5:**
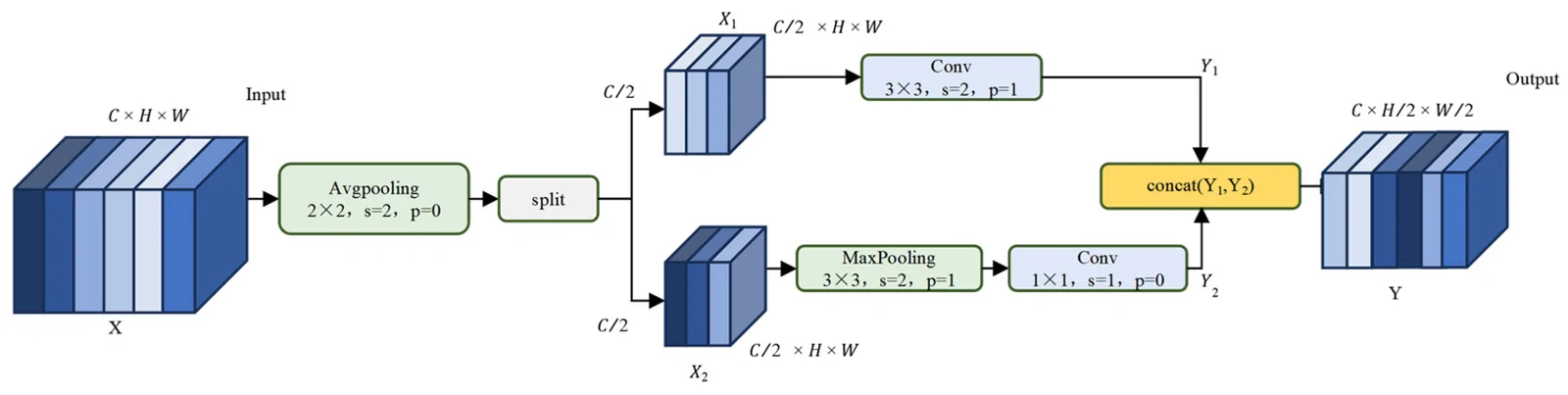
Structure of the ADown lightweight downsampling module. *X* is the input feature map, *X*_1_ and *X*_2_ are the two branch features obtained by channel-wise split, *Y*_1_ is the output of the upper branch convolution, *Y*_2_ is the output of the lower branch pooling and convolution, *Y* is the output feature of the module; *C* is the number of channels in the feature map, *H* and *W* are the height and width of the feature map, s denotes the stride of pooling, and p denotes the padding of convolution.

In detection networks, downsampling decreases feature-map size and enlarges the receptive field. However, stride convolution may remove boundary details and small-target cues during compression. Cow heads in inspection images have variable scales, may be partially hidden by fences, and can be affected by strong illumination. ADown is therefore placed at selected downsampling positions. As illustrated in Figure 5, the input feature first passes through 2 × 2 average pooling and is then split by channel. One branch performs local extraction with 3 × 3 convolution, while the other applies 3 × 3 max pooling and 1 × 1 convolution for spatial compression and channel adjustment. Concatenating the two outputs retains spatial cues from different operations and reduces the computational burden for subsequent detection.

#### 2.2.3. Construction of the C3k2_FE Feature-Adaptive Enhancement Module

**Figure 6:**
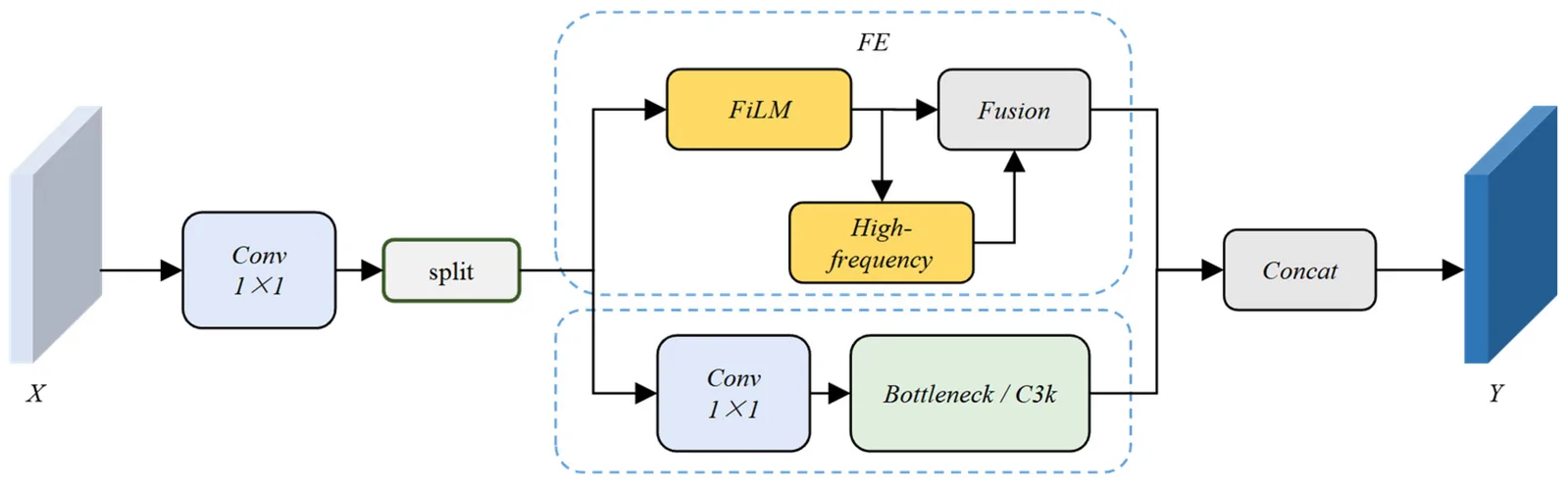
Structure of the FiLM-Enhanced C3k2_FE Module. *X* represents the input feature of the C3k2_FE module, and *Y* represents the output feature. FE denotes the feature-adaptive enhancement module, FiLM denotes feature-wise linear modulation, and High-frequency denotes the high-frequency residual enhancement branch.

The two imaging modalities provide complementary but inconsistent information. Visible images contain rich edges and local textures, but their quality changes with illumination. Infrared pseudo-colour images encode thermal radiation and are less affected by visible light, yet they usually lack fine texture. Such modality differences can reduce the generalization of a detector trained on mixed dual-light data. To address this issue, the C3k2_FE module, C3k2 with FiLM Enhancement, is inserted into the high-level feature-extraction part of the backbone and the multi-scale reconstruction part of the neck, as shown in Figure 6.

**Figure 7:**
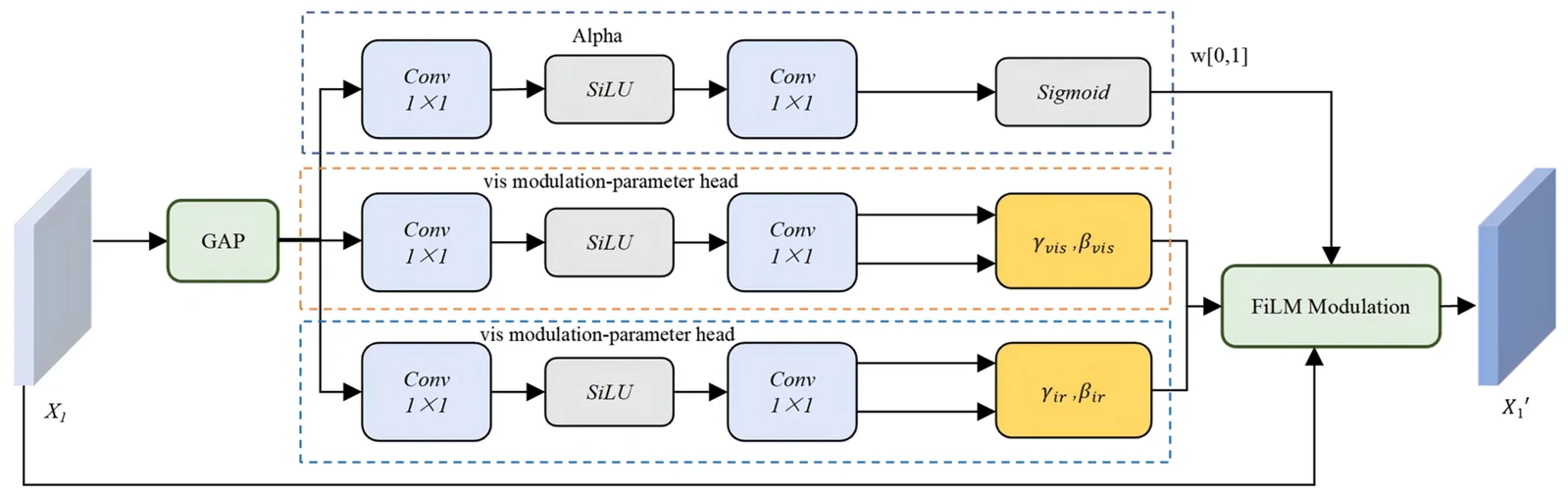
Structure of the FiLM branch in the C3k2_FE module. *X*_1_ is the input feature; GAP denotes global average pooling; Alpha, vis head, and ir head represent the branches generating the learnable interpolation weight, visible-light affine parameters, and infrared affine parameters, respectively; w is the learnable interpolation weight; *X*_1_’ is the output feature after channel-wise FiLM modulation.

C3k2_FE augments the original C3k2 block with a FiLM branch (Figure 7). A global descriptor is first obtained from the input feature by global average pooling. This descriptor is then fed into three lightweight branches: an Alpha branch that predicts interpolation weights and two FiLM branches that generate modality-related affine parameters. Each FiLM branch uses two consecutive 1 × 1 convolutions with SiLU activation. The first convolution compresses the channel dimension for inexpensive transformation, and the second outputs twice the input-channel number, which is separated into scale and shift terms. The Alpha branch uses 1 × 1 convolutions, SiLU, and Sigmoid to constrain the interpolation weights to [0, 1]. The two sets of affine parameters are blended with these weights and applied to the input feature along the channel dimension. Because the modulation is inferred from global feature statistics rather than explicit modality labels, the block can adjust channel responses adaptively and reduce fluctuations caused by cross-modal differences.

(1) $$G=G\mathrm{lobalAvgPool}2d(X),\;G\in R^{C\times 1\times 1}$$

(2a) $$\left(\gamma_{\mathrm{vis}\;},\beta_{\mathrm{vis}}\right)={\mathrm{Conv}}_{\mathrm{vis}}(G)$$

(2b) $$\left(\gamma_{\mathrm{ir}\;},\beta_{\mathrm{ir}}\right)={\;\mathrm{Conv}}_{\mathrm{ir}}(G)$$

(3a) $$\gamma =w\cdot \gamma_{\mathrm{vis}\;}+(1-w)\cdot \gamma_{\mathrm{ir}\;}$$

(3b) $$\beta =w\cdot \beta_{\mathrm{vis}\;}+(1-w)\cdot \beta_{\mathrm{ir}}$$

(4) $${X_{1}}^{\prime }=\gamma ⊙X+\beta$$

where *G* ∈ **ℝ**^C×1×1^ represents the channel-wise context vector after global pooling, *γ_vis_*, *β_vis_*, *γ_ir_*, *β_ir_* denote the affine parameters generated by the visible-light and infrared branches, *w* is the learnable interpolation coefficient, ${X_{1}}^{\prime }$ is the channel-modulated output feature, and ⊙ represents channel-wise element-wise multiplication.

**Figure 8:**
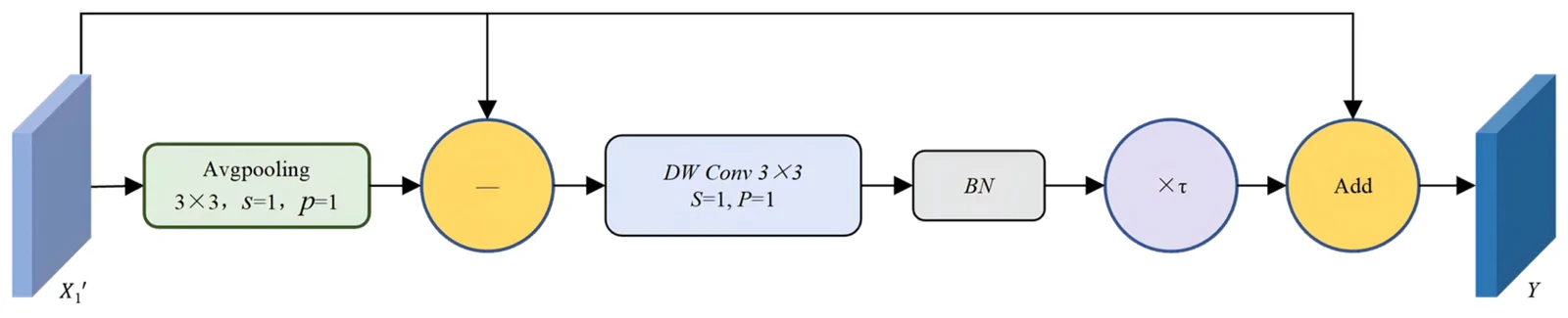
Structure of the high-frequency residual enhancement branch. ${X_{1}}^{\prime }$ is the input feature after FiLM modulation; Y is the output feature after high-frequency residual enhancement.

A high-frequency residual branch is also added to emphasize detailed cow-head structures (Figure 8). This branch obtains a low-frequency component through local 3 × 3 average pooling and subtracts it from the FiLM-modulated feature to isolate high-frequency information. The residual is refined with 3 × 3 depthwise convolution and batch normalization, and is then added back to the FiLM-modulated feature while preserving the output-channel number. A learnable scaling factor controls the contribution of the residual and is optimized during training through the detection loss. In this way, the model can selectively strengthen edges, contours, and local texture cues in scenes with harsh illumination, weak infrared texture, or occlusion.

(5) $$X_{\mathrm{low}}={\;\mathrm{AvgPool}}_{3\times 3,s=1,p=1}\left({X_{1}}^{\prime }\right)$$

(6) $$X_{\mathrm{hf}\;}={X_{1}}^{\prime }-X_{\mathrm{low}}$$

(7) $${\hat{X}}_{\mathrm{hf}\;}=\mathrm{BN}\left({\mathrm{DWConv}}_{3\times 3}\left(X_{\mathrm{hf}}\right)\right)$$

(8) $$Y={X_{1}}^{\prime }+\tau \cdot {\hat{X}}_{\mathrm{hf}}$$

where ${X_{1}}^{\prime }$ denotes the feature after FiLM modulation; $X_{\mathrm{low}}$ denotes the low-frequency component extracted by 3 × 3 average pooling; $X_{\mathrm{hf}}$ denotes the high-frequency residual; ${\mathrm{DWConv}}_{3\times 3}$ denotes the 3 × 3 depthwise convolution; BN(·) denotes the batch normalization operation; ${\hat{X}}_{\mathrm{hf}}$ denotes the high-frequency feature enhanced by depthwise convolution and batch normalization; τ denotes the learnable scaling coefficient; and *Y* denotes the output feature obtained by fusing the high-frequency residual enhancement branch with the FiLM-modulated feature.

#### 2.2.4. SPPF_ECA Multi-Scale Channel Attention Enhancement Module

**Figure 9:**
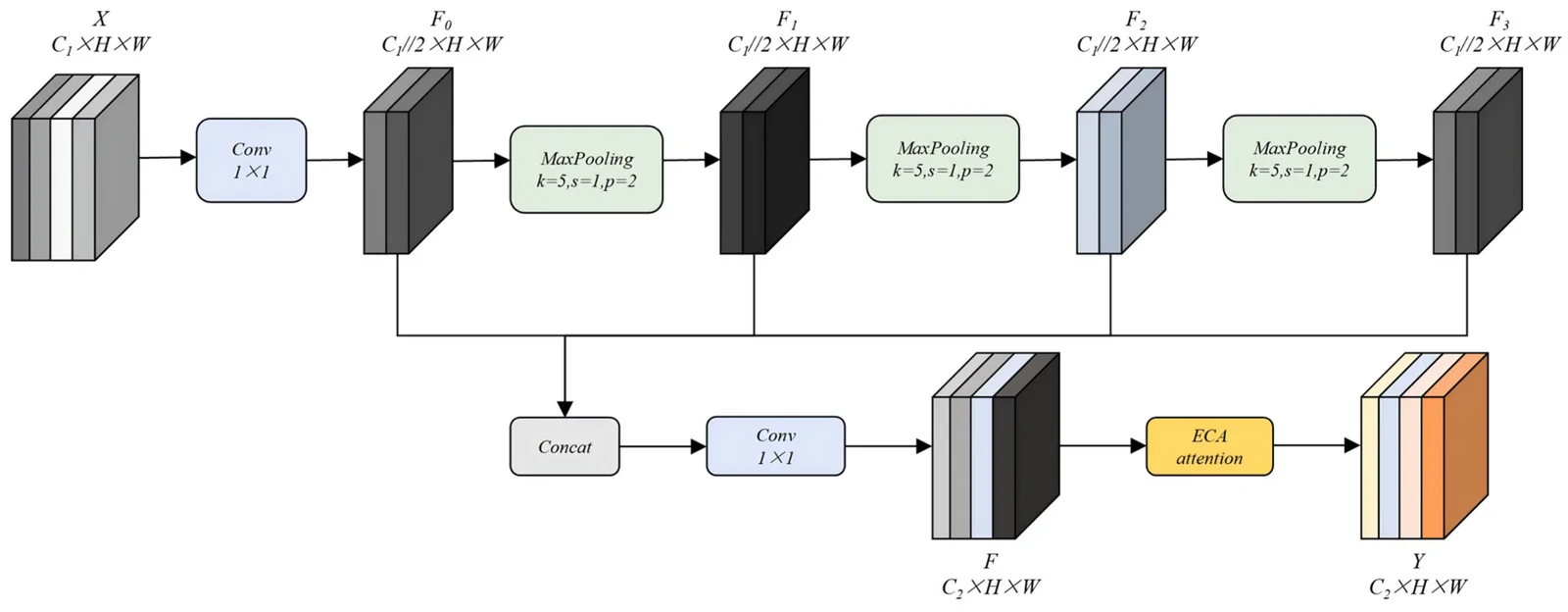
Structure of the SPPF_ECA multi-scale channel attention module. X denotes the input feature of the SPPF_ECA module; *F*_0_, *F*_1_, *F*_2_ and *F*_3_ denote the multi-scale features obtained by 1 × 1 convolution and successive max-pooling operations; *F* denotes the SPPF output feature after feature concatenation and 1 × 1 convolution fusion; Y denotes the output feature recalibrated by ECA channel attention.

SPPF obtains multi-scale semantics by repeated max pooling and increases the receptive field with little extra computation. Its operation, however, mainly fuses information in the spatial domain and does not explicitly reweight channels. In dual-light cow-head detection, visible and infrared inputs activate different informative channels, so spatial pyramid pooling alone may not sufficiently emphasize cow-head-related responses. Therefore, ECA is appended after SPPF to produce the SPPF_ECA module shown in Figure 9.

**Figure 10:**
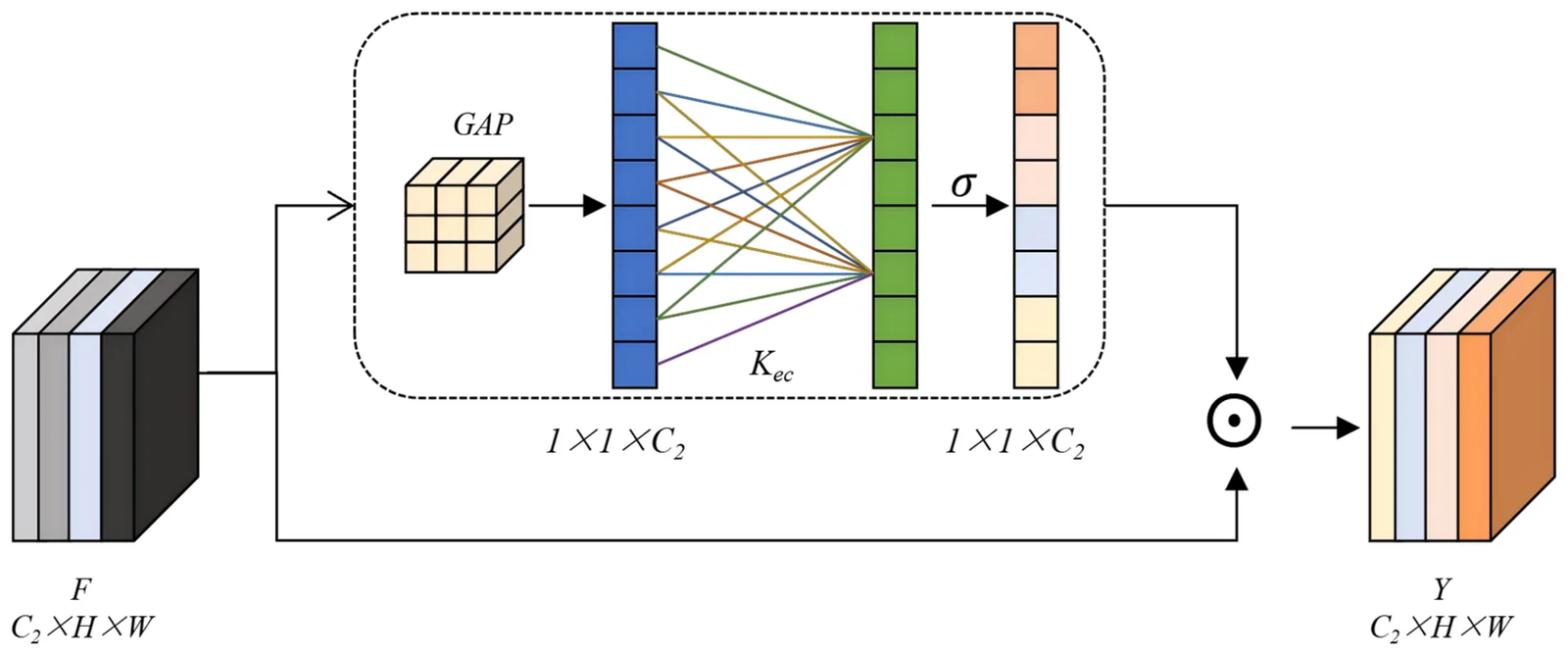
Structure of the Efficient Channel Attention (ECA) module. *GAP* denotes global average pooling; *Kec* denotes the kernel size of the 1D convolution in ECA; *σ* denotes the Sigmoid activation function; ⊙ denotes element-wise multiplication.

As shown in Figure 10, in ECA, each channel is first compressed to a scalar by adaptive global average pooling, yielding a compact descriptor:

(9) $$Z_{c}\;=\;\mathrm{AdaptiveAvgPool}2d\left(F_{c}\right),\;\forall c\;\in \{1,2,\ldots ,C\}$$

where $F_{c}$ denotes the feature map of the c channel.

Neighbouring-channel interactions are then modelled with a one-dimensional convolution whose kernel size is chosen from the channel number. Sigmoid activation converts the response into channel weights:

(10) $$K_{\mathrm{ec}}\;={\left|\frac{\log_{2}(C)+b}{\gamma }\right|}_{\mathrm{odd}}$$

(11) $$s=\sigma \left({Conv1D}_{k_{ec}}(z)\right)$$

where C denotes the number of input channels, *b* = 1 and *γ* = 2 are the tuning factors, and ${\left|\cdot \right|}_{\mathrm{odd}}$ denotes the nearest odd integer [28]. The rounding operation ensures an odd kernel size for centre alignment, the Sigmoid function normalizes the response, and the resulting vector stores channel-wise weights.

The feature map is multiplied by these weights to obtain the recalibrated output:

(12) $$F^{\prime }\;=\;F\;⨀s$$

where ⨀ denotes channel-wise element-wise multiplication.

Unlike channel-attention designs that compress channels with fully connected layers, ECA keeps the channel dimension unchanged and adds little computation. It therefore improves important channel responses without compromising lightweight inference. In the proposed network, SPPF_ECA enhances high-level cow-head semantics and improves discrimination from complex backgrounds.

### 2.3. Improvement of the Heterogeneous-Camera Target Association Algorithm

In a moving robot system, the visible and infrared cameras are installed at different positions and differ in resolution and field of view. The same cow head may therefore appear at different scales, positions, and visual forms in the two modalities. Animal movement, occlusion by fences, pose changes, and missed detections can also make the target numbers inconsistent between synchronized image pairs. Pixel-level matching is unsuitable in this setting because it is sensitive to the appearance gap between texture images and thermal pseudo-colour images and because it introduces a large computational burden for edge processing.

The association module uses the cow-head detection boxes produced by YOLO11-AFE as input. Rather than aligning the two images at the pixel level, it constructs association cues from the overlap and normalized relative spatial positions of the detection boxes. The task considered in this study is instantaneous target-level association within a single synchronized visible-light–infrared image pair. It does not estimate a whole-image geometric transformation, determine persistent animal identities, or rely on temporal trajectories. Because differences in field of view, occlusion, and missed detections may produce unequal target numbers, the method establishes a partial one-to-one association and permits detections without valid counterparts to remain unassociated. This bounding-box-based formulation maintains low computational complexity and is suitable for deployment on embedded robotic platforms.

#### 2.3.1. Problem Definition and Overall Workflow

YOLO11-AFE outputs two bounding-box sets from synchronized visible-light and infrared pseudo-colour frames. They are defined as follows:

(13a) $$B^{\mathrm{vis}}\;=\;\left\{b_{1}^{\mathrm{vis}},\;b_{2}^{\mathrm{vis}},\;\ldots ,b_{m}^{\mathrm{vis}}\right\}$$

(13b) $$B^{\mathrm{ir}}=\left\{b_{1}^{\mathrm{ir}},\;b_{2}^{\mathrm{ir}},\;\ldots ,b_{n}^{\mathrm{ir}}\right\}$$

where $B^{\mathrm{vis}}$ and $B^{\mathrm{ir}}$ denote the sets of cow-head bounding boxes in the visible-light and infrared pseudo-colour images, respectively; (m) and (n) denote the numbers of cow-head targets detected in the visible-light and infrared pseudo-colour images, respectively; and $b_{m}^{\mathrm{vis}}$ and $b_{n}^{\mathrm{ir}}$ denote the i-th visible-light cow-head bounding box and the j-th infrared pseudo-colour cow-head bounding box, respectively.

Because occlusion, missed detection, and camera field-of-view differences can make the two target counts unequal, the goal is not to pair every detection blindly. Instead, the task is to obtain the most plausible correspondence between the visible and infrared box sets:

(14) $$M=\left\{\left(b_{i}^{\mathrm{vis}},b_{j}^{\mathrm{ir}}\right)|i\in [1,m],j\in [1,n]\right\}$$

where *M* denotes the set of matched cow-head targets between the visible-light and infrared pseudo-colour images.

If a pair is accepted, the two boxes are treated as observations of the same cow head; otherwise, the candidate is discarded as unmatched. The proposed workflow first builds a cost matrix from two spatial cues: box overlap and the relative distance of each box centre to the corresponding image centre. The Hungarian algorithm then searches for the minimum-cost assignment, and additional thresholds remove abnormal pairs. This procedure yields one-to-one correspondence for multi-cow dual-light scenes.

#### 2.3.2. Construction of the Fused Cost Matrix

A cow head captured by the two cameras usually preserves approximate relative spatial consistency, but its absolute pixel coordinates differ because the sensors have different resolutions, views, and mounting positions. Directly measuring the pixel distance between two box centres can therefore be misleading. To reduce sensitivity to these factors, each box centre is compared with the centre of its own image, and the difference between the two centre-distance values is used as a spatial consistency term. The IoU of the two boxes is then incorporated to represent regional overlap, and the two measurements jointly form the fused cost matrix.

For a visible-light candidate and an infrared candidate, their box-centre coordinates are written as:

(15a) $$p_{i}^{\mathrm{vis}}\;=\;\left(x_{i}^{\mathrm{vis}},{\;y}_{i}^{\mathrm{vis}}\right)$$

(15b) $$p_{j}^{\mathrm{ir}}=\left(x_{j}^{\mathrm{ir}},\;y_{j}^{\mathrm{ir}}\right)$$

The centres of the corresponding visible-light and infrared pseudo-colour images are defined as:

(16a) $$o^{\mathrm{vis}}\;=\;\left(x_{0}^{\mathrm{vis}},y_{0}^{\mathrm{vis}}\right)$$

(16b) $$o^{\mathrm{ir}}=\left(x_{0}^{\mathrm{ir}},y_{0}^{\mathrm{ir}}\right)$$

The distance from the visible-light box centre to the visible-light image centre is calculated by:

(17) $$d_{i}^{\;\mathrm{vis}}\;=\;\sqrt{{\left(x_{i}^{\mathrm{vis}}-x_{0}^{\mathrm{vis}}\right)}^{2}+{\left(y_{i}^{\mathrm{vis}}-y_{0}^{\mathrm{vis}}\right)}^{2}}$$

The same calculation is performed for the infrared pseudo-colour box centre and its image centre:

(18) $${d\;}_{j}^{\mathrm{ir}}\;=\;\sqrt{{\left(x_{j}^{\mathrm{ir}}-x_{0}^{\mathrm{ir}}\right)}^{2}+{\left(y_{j}^{\mathrm{ir}}-y_{0}^{\mathrm{ir}}\right)}^{2}}$$

The relative location discrepancy of the two candidates is then expressed by the normalized difference between these two distances:

(19) $$D_{\mathrm{ij}}\;=\;\frac{\left|d_{i}^{\mathrm{vis}}-d_{j}^{\mathrm{ir}}\right|}{d_{max}}$$

where $p_{i}^{\mathrm{vis}}$ and $p_{j}^{\mathrm{ir}}$ denote the centre coordinates of the visible-light and infrared pseudo-colour detection boxes, respectively; $o^{\mathrm{vis}}$ and $o^{\mathrm{ir}}$ denote the centre coordinates of the visible-light and infrared pseudo-colour images, respectively; $d_{i}^{\mathrm{vis}}$ and $d_{j}^{\mathrm{ir}}$ denote the Euclidean distances from the detection-box centres to the corresponding image centres; $D_{\mathrm{ij}}$ denotes the normalized relative centre-distance difference; and $d_{max}$ is the normalization scale factor, which can be set as the diagonal length of the corresponding image or the maximum possible distance difference. A smaller $D_{\mathrm{ij}}$ indicates that the two targets have more similar relative spatial positions in their respective images.

IoU is used to measure how much the two candidate boxes overlap:

(20) $${\mathrm{IoU}}_{\mathrm{ij}}\;=\;\frac{b_{i}^{\mathrm{vis}}\cap b_{j}^{\mathrm{ir}}}{b_{i}^{\mathrm{vis}}\cup b_{j}^{\mathrm{ir}}}$$

where ${\mathrm{IoU}}_{\mathrm{ij}}$ denotes the intersection over union between the i-th visible-light detection box and the j-th infrared pseudo-colour detection box; $b_{i}^{\mathrm{vis}}\cap b_{j}^{\mathrm{ir}}$ denotes the intersection area of the two detection boxes; and $b_{i}^{\mathrm{vis}}\cup b_{j}^{\mathrm{ir}}$ denotes their union area. A larger ${\mathrm{IoU}}_{\mathrm{ij}}$ indicates a higher degree of spatial overlap between the two detection boxes.

The matching cost for a visible-light target and an infrared pseudo-colour target is then defined by combining the normalized centre-distance difference and the IoU term:

(21) $$C_{\mathrm{ij}\;}\;=\;\lambda D_{\mathrm{ij}}+(1-\lambda )\left(1-{\mathrm{IoU}}_{\mathrm{ij}}\right)$$

where $C_{\mathrm{ij}}$ denotes the matching cost of the target pair ($b_{i}^{\mathrm{vis}},b_{j}^{\mathrm{ir}}$); λ is the weighting coefficient used to balance the effects of the relative centre-distance difference and IoU on the matching cost; and $1-{\mathrm{IoU}}_{\mathrm{ij}}$ is the overlap penalty term. *A* smaller $C_{\mathrm{ij}}$ indicates a higher probability that the visible-light target and the infrared pseudo-colour target correspond to the same cow head.

After the pairwise costs are obtained, they are arranged into the heterogeneous-camera association matrix:

(22) $$C=\left[\begin{array}{cccc} C_{11} & C_{12} & \ldots & C_{1n}\\ C_{21} & C_{22} & \ldots & C_{2n}\\ \vdots & \vdots & \ddots & \vdots \\ C_{m1} & C_{m2} & \ldots & C_{\mathrm{mn}} \end{array}\right]$$

where C denotes the heterogeneous-camera target association cost matrix with a dimension of *m* × *n*, and each element represents the matching cost between one visible-light target and one infrared pseudo-colour target.

#### 2.3.3. Improved Hungarian Matching and Outlier Association Removal

Given the fused cost matrix, the Hungarian algorithm is adopted to find the minimum-cost one-to-one assignment between visible-light and infrared pseudo-colour detections. This global optimization avoids the duplicated or incorrect matches that may occur with local nearest-neighbour rules [29]. The objective is:

(23) $$M^{*}=\arg\underset{M}{\min}\sum_{(i,j)\in M}C_{\mathrm{ij}}$$

where $M^{*}$ denotes the set of optimal matches, *M* denotes all feasible match combinations, and $C_{\mathrm{ij}}$ represents the association cost between the i-th visible-light target and the j-th infrared pseudo-colour target.

In real inspection data, some detections should not be assigned because the two cameras may capture different target sets. If every Hungarian output were retained, boxes with large relative displacement or negligible overlap could be incorrectly forced into pairs. For this reason, threshold filtering is applied after assignment. A candidate match is kept only when:

(24) $$C_{\mathrm{ij}}<T_{c}$$

with additional constraints on relative centre-distance difference and IoU:

(25) $$D_{\mathrm{ij}}<T_{d}\;,\;{\mathrm{IoU}}_{\mathrm{ij}}>T_{\mathrm{iou}}$$

where $T_{c}$ is the cost threshold, $T_{d}$ is the relative centre-distance threshold, and $T_{\mathrm{iou}}$ is the IoU threshold.

Candidates violating any threshold are discarded, giving the valid matching set:

(26) $$M_{\mathrm{valid}}\;=\;\{(b_{i}^{\mathrm{vis}},b_{j}^{\mathrm{ir}})|C_{\mathrm{ij}}<T_{c},\;D_{\mathrm{ij}}<T_{d},\;{\mathrm{IoU}}_{\mathrm{ij}}>T_{\mathrm{iou}}\}$$

where $M_{\mathrm{valid}}$ denotes the set of matches retained after outlier removal.

At inference, the association algorithm operates on model detections rather than annotation labels. Given *m* visible-light detections and *n* infrared detections, the rectangular cost matrix permits unequal target numbers. When *m ≠ n*, only a subset of detections can enter the one-to-one assignment, and surplus detections remain unassigned. After the initial Hungarian assignment, candidate pairs that do not satisfy the cost, relative centre-distance, and IoU constraints are removed. Therefore, a valid visible-light cow-head detection without a reliable infrared counterpart is retained as an unassociated visible-light target rather than being forcibly paired with an unrelated infrared detection. Only valid cross-camera associations are forwarded to subsequent local registration and temperature-extraction procedures.

The post-assignment thresholds were set to *T_c_* = 1.00, *T_d_* = 0.30, and *T_IoU_* = 0.05. A candidate association was retained only when its fused cost was lower than *T_c_*, its normalized centre-distance value was lower than *T_d_*, and its IoU was greater than *T_IoU_*. These threshold values were kept unchanged in all subsequent target-association experiments.

In summary, the association method uses YOLO11-AFE detections as its input and constructs a lightweight cost matrix from centre-distance discrepancy and IoU. The improved Hungarian matching then provides one-to-one correspondence between visible-light and infrared pseudo-colour cow-head targets. Because no pixel-level cross-modal registration is required, the method remains efficient and can handle occlusion, target overlap, and unequal target counts while supplying target-level correspondence for future non-contact facial-temperature monitoring in dairy cows.

### 2.4. Hardware Environment and Evaluation Metrics

The experiments were run on a server equipped with an NVIDIA RTX A6000 GPU (NVIDIA Corporation, Santa Clara, CA, USA). The software stack consisted of Ubuntu 20.04.6 LTS, Python 3.10.19, PyTorch 2.9.1, and CUDA 12.8.

For training, images were resized to 640 × 640 pixels, the batch size was 64, and 200 epochs were scheduled with 8 data-loading workers. Stochastic gradient descent was used for optimization. To assess the reproducibility of the results, each model and ablation configuration was independently trained three times using random seeds 523, 321, and 2026 under otherwise identical experimental settings. The reported results are presented as the mean ± sample standard deviation across the three runs. To stabilize training and reduce overfitting, early stopping was enabled with patience set to 50.

Model accuracy was reported with precision (P), mAP at an IoU threshold of 0.5, and mAP averaged over IoU thresholds from 0.5 to 0.95. Computational complexity was described using the number of trainable parameters and GFLOPs. The following equations define these metrics:

(27) $$P\;=\;\frac{\mathrm{TP}}{\mathrm{TP}+\mathrm{FP}}$$

(28) $$\mathrm{AP}=\int_{0}^{1}P(R)dR$$

(29) $$\mathrm{mAP}=\frac{1}{N}\sum_{i=1}^{N}AP_{i}$$

where TP denotes the number of true positive detections, FP denotes the number of false-positive detections, P(R) represents the precision–recall curve, AP denotes the average precision for a single class, $AP_{i}$ denotes the average precision of the i-th class, and N is the total number of target classes.

## 3. Results

### 3.1. Comparison of Detection Models

Cow-head detection was evaluated separately for the visible-light and infrared pseudo-colour test subsets. YOLO11-AFE was benchmarked against RT-DETR_L, Faster-RCNN, YOLOv5n, YOLOv8n, YOLOv10n, YOLO11n, and YOLOv12n. To ensure an unbiased comparison, all models were trained again with the same data split, input resolution, and training configuration before being tested on both modalities. The results are given in Table 2.

As Table 2 indicates, YOLO11-AFE achieved the highest mean detection performance on both the visible-light and infrared pseudo-colour test subsets. All results are reported as the mean ± sample standard deviation of three independent runs using random seeds 523, 321, and 2026. On the visible-light subset, YOLO11-AFE achieved a Precision of 97.590 ± 0.2022%, an mAP@0.5 of 96.373 ± 0.2409%, and an mAP@0.5:0.95 of 70.757 ± 0.5052%. Compared with the strongest competing results, its Precision and mAP@0.5 exceeded those of YOLOv10n by 1.110 and 0.506 percentage points, respectively, while its mAP@0.5:0.95 was 0.784 percentage points higher than that of YOLOv8n. On the infrared pseudo-colour subset, YOLO11-AFE achieved a Precision of 96.113 ± 0.2386%, an mAP@0.5 of 99.073 ± 0.0950%, and an mAP@0.5:0.95 of 91.500 ± 0.4026%. Its Precision was 0.273 percentage points higher than that of YOLOv5n, while its mAP@0.5 and mAP@0.5:0.95 exceeded those of YOLO11n and YOLOv10n by 0.290 and 1.047 percentage points, respectively.

Compared with the YOLO11n baseline, YOLO11-AFE increased the visible-light Precision, mAP@0.5, and mAP@0.5:0.95 by 4.743, 2.240, and 1.554 percentage points, respectively. On the infrared pseudo-colour subset, the corresponding improvements were 1.846, 0.290, and 1.157 percentage points. Across the six modality-specific metrics, the standard deviations of YOLO11-AFE ranged from 0.0950 to 0.5052 percentage points. Moreover, YOLO11-AFE produced higher values than YOLO11n for all six metrics under each of the three corresponding random seeds, demonstrating that the observed improvements were consistent across independent training runs rather than being determined by a single random initialisation.

Meanwhile, the parameter count decreased from 2.59 M to 2.14 M, and the FLOPs decreased from 6.44 G to 5.27 G, corresponding to reductions of 17.37% and 18.17%, respectively. YOLO11-AFE also required substantially fewer computational resources than RT-DETR-L and Faster R-CNN while achieving higher mean detection accuracy on both modalities. These results demonstrate that the proposed improvements enhance visible–thermal cow-head detection while reducing model complexity, thereby providing a favourable accuracy–efficiency balance for deployment on resource-constrained robotic inspection platforms.

**Figure 11:**
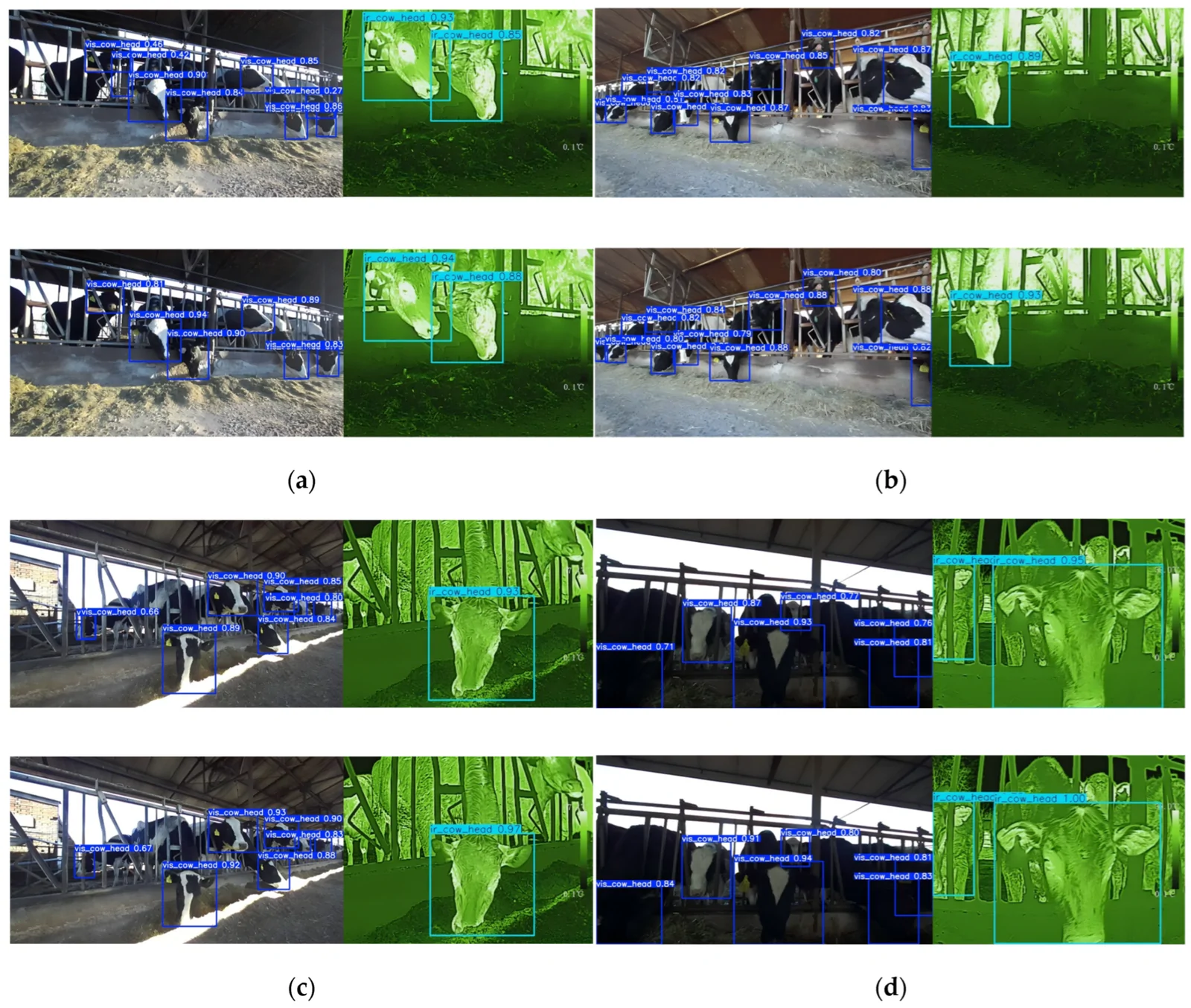
Qualitative comparison of cow-head detection results between YOLO11n and YOLO11-AFE under different complex dairy-farm scenes. In each panel, the upper images show the results of YOLO11n, and the lower images show the results of YOLO11-AFE. (**a**) Strong-light interference scene; (**b**) normal illumination scene; (**c**) local shadow interference scene; (**d**) low-illumination scene.

Four difficult farm conditions were selected for qualitative comparison with YOLO11n: strong-light interference, dense multi-cow distribution, local shadow, and low illumination. Figure 11 shows that YOLO11-AFE gives more stable cow-head locations and higher confidence scores. The improvement is consistent with the roles of the three modules: ADown alleviates information loss in downsampling, C3k2_FE improves adaptation between visible and infrared appearances and enhances fine details, and SPPF_ECA reweights important high-level channels. These visual results support the quantitative finding that the model is more robust across challenging dual-light farm scenes.

### 3.2. Ablation Study Analysis

Table 3 presents the ablation results obtained from three independent runs using random seeds 523, 321, and 2026. The effects of the proposed modules varied across the two imaging modalities and evaluation metrics, indicating that increasing the number of modules did not necessarily produce monotonic improvements in every metric. When ADown was introduced independently, the visible-light Precision increased from 92.847 ± 0.3496% to 97.187 ± 0.2021%, while the infrared Precision increased from 94.267 ± 0.5150% to 95.770 ± 0.1562%, corresponding to improvements of 4.340 and 1.503 percentage points, respectively. Meanwhile, the parameter count decreased from 2.59 M to 2.04 M and the FLOPs decreased from 6.44 G to 5.17 G, representing reductions of 21.24% and 19.72%, respectively. The marked improvement in Precision indicates that ADown improved the discrimination between cow heads and background interference and reduced the relative occurrence of false-positive detections. This result is consistent with its dual-branch downsampling structure: average pooling suppresses local fluctuations before channel splitting, whereas the convolutional and max-pooling branches retain complementary local and salient spatial responses. Their concatenation therefore preserves useful cow-head boundary and structural cues while limiting responses to background elements such as fences, illumination artefacts, and thermal-background variations. The smaller changes in mAP@0.5 than in Precision suggest that ADown mainly reduced false-positive detections at the selected confidence threshold, while its effect on the overall precision–recall performance was more moderate.

The individual-module experiments also revealed different functional emphases. C3k2_FE produced the highest visible-light mAP@0.5:0.95 of 70.860 ± 0.3617% when used independently and increased the infrared mAP@0.5:0.95 to 91.477 ± 0.1124%, indicating an improvement in feature representation and localization under stricter IoU thresholds. SPPF_ECA increased the visible-light mAP@0.5 to 95.737 ± 0.0751% and the infrared mAP@0.5 to 98.940 ± 0.1735%, but its effect on Precision was modality dependent. The paired-module configurations further demonstrate that the interactions among the modules were metric- and modality-specific rather than uniformly complementary. ADown+C3k2_FE achieved the highest infrared Precision of 96.867 ± 0.4302%, but its visible-light mAP@0.5:0.95 was lower than that obtained using C3k2_FE alone. Similarly, C3k2_FE+SPPF_ECA maintained high visible-light detection performance but reduced infrared Precision to 93.893 ± 0.0643%. The Adown + SPPF_ECA configuration produced lower values than both of its corresponding single-module configurations across all six modality-specific metrics. These results indicate that changes in downsampling, adaptive feature representation, and channel recalibration alter the balance between false-positive suppression, confidence ranking, and bounding-box localization, and their effects are not simply additive.

The complete YOLO11-AFE configuration was selected on the basis of its overall accuracy–complexity balance rather than its superiority in every individual metric. It achieved the highest mean visible-light Precision and mAP@0.5, reaching 97.590 ± 0.2022% and 96.373 ± 0.2409%, respectively, together with the highest infrared mAP@0.5 and mAP@0.5:0.95 of 99.073 ± 0.0950% and 91.500 ± 0.4026%. Its visible-light mAP@0.5:0.95 was 70.757 ± 0.5052%, only 0.103 percentage points below the highest value obtained using C3k2_FE alone, while its infrared Precision was 96.113 ± 0.2386%, 0.754 percentage points below that of ADown+C3k2_FE. Compared with the C3k2_FE-only configuration, the complete model reduced the parameter count and FLOPs by 18.32% and 18.42%, respectively, while improving visible-light Precision and mAP@0.5 and maintaining comparable strict-IoU performance. Relative to the YOLO11n baseline, the complete model improved the visible-light Precision, mAP@0.5, and mAP@0.5:0.95 by 4.743, 2.240, and 1.554 percentage points, respectively, and improved the corresponding infrared metrics by 1.846, 0.290, and 1.157 percentage points. At the same time, its parameter count and FLOPs were reduced by 17.37% and 18.17%. Therefore, the complete configuration was retained because it provided the most balanced performance across both modalities while preserving a lightweight architecture suitable for edge deployment.

### 3.3. Heterogeneous-Camera Target Association Results Analysis

The heterogeneous-camera target-association method was evaluated using the synchronized visible-light and infrared pseudo-colour image pairs in the validation and test subsets. YOLO11-AFE first produced cow-head detection boxes for the two modalities. The association algorithm subsequently constructed a fused cost matrix using IoU and the normalized relative centre-distance difference between candidate targets, followed by global one-to-one assignment and abnormal-association removal. The results for the validation and test subsets are reported separately in Table 4, together with an overall descriptive summary.

The target association accuracy is computed as:

(30) $${\mathrm{Acc}}_{\mathrm{match}}\;=\;\frac{N_{\mathrm{correct}}}{N_{\mathrm{total}}}\times 100\%$$

where ${\mathrm{Acc}}_{\mathrm{match}}$ denotes the target association accuracy, $N_{\mathrm{correct}}$ represents the number of correctly matched cow-head target pairs, and $N_{\mathrm{total}}$ is the total number of cow-head target pairs determined from manual annotation.

For association evaluation, $N_{\mathrm{total}}$ included only manually confirmed cow-head pairs with valid annotations in both modalities. Visible-only or infrared-only annotations were not counted as missed associations because no valid cross-camera counterpart existed. However, if the algorithm incorrectly assigned such a detection to an unrelated target in the other modality, the result was counted as an incorrect association.

The validation subset contained 122 synchronized image pairs and 184 manually confirmed cow-head pairs. Among them, 177 pairs were correctly associated, six were incorrectly associated, and one pair remained unassociated, resulting in an association accuracy of 96.20%. The test subset contained 122 synchronized image pairs and 187 cow-head pairs. All 187 pairs were correctly associated, yielding an association accuracy of 100.00%. Across the two evaluation subsets, 364 of the 371 ground-truth cow-head pairs were correctly associated, six were incorrectly associated, and one remained unassociated. The combined association accuracy was 98.11%.

To examine the influence of the three post-assignment thresholds on target-association performance, a one-factor-at-a-time sensitivity analysis was conducted by varying each threshold individually while holding the other two at their selected values. The results showed that overly strict cost and centre-distance thresholds, as well as an excessively high IoU threshold, reduced the number of retained correct associations. The selected thresholds provided stable association performance within the evaluated ranges. The complete sensitivity-analysis results are provided in Table S3 in the Supplementary Materials.

**Figure 12:**
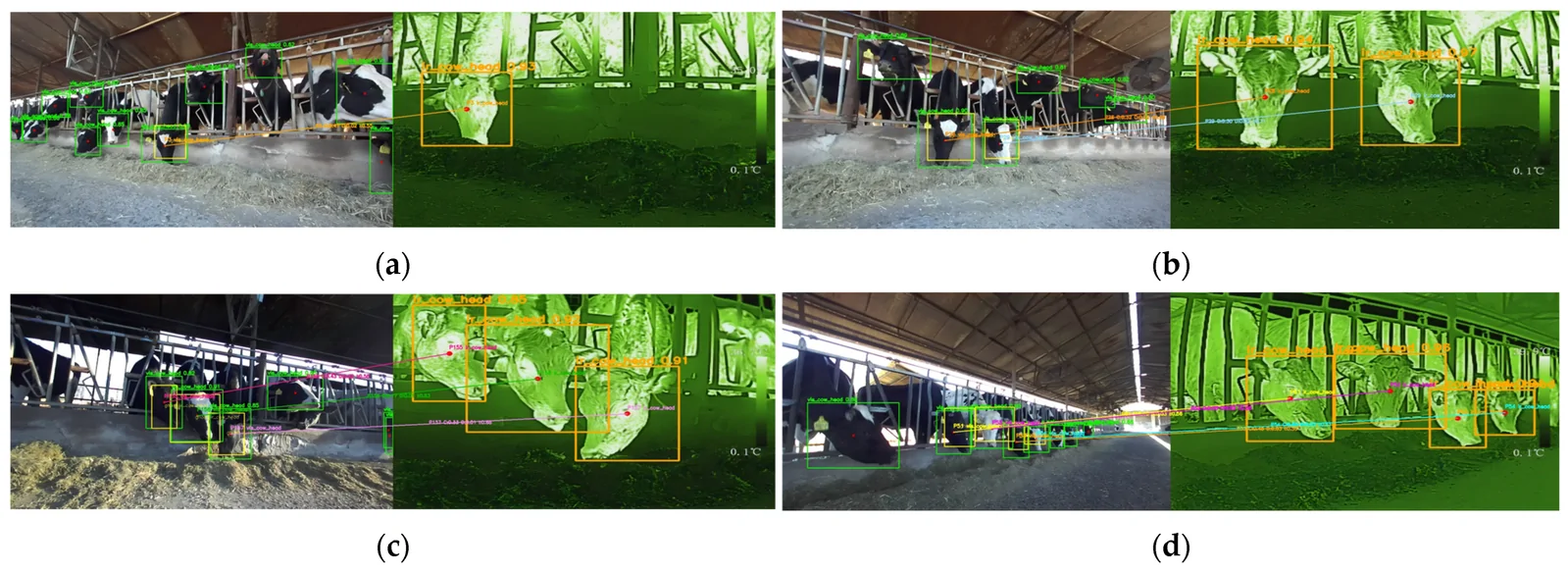
Visualization results of cow-head target association between heterogeneous cameras. (**a**) Association result in a single-target scene; (**b**) Association result in a two-target scene; (**c**) Association result in a three-target scene; (**d**) Association result in a four-target scene.

Figure 12 shows examples of heterogeneous-camera association. Lines connect boxes that correspond to the same cow across the visible-light and infrared pseudo-colour views. The examples demonstrate that the algorithm can maintain cross-modal matching in multi-cow scenes and under spatial offset. Matches that do not pass the constraints are removed from the final set, which avoids erroneous forced associations. This provides a dependable basis for subsequent local registration, facial-region recognition, and radiometric temperature extraction at the same inspection moment.

**Figure 13:**
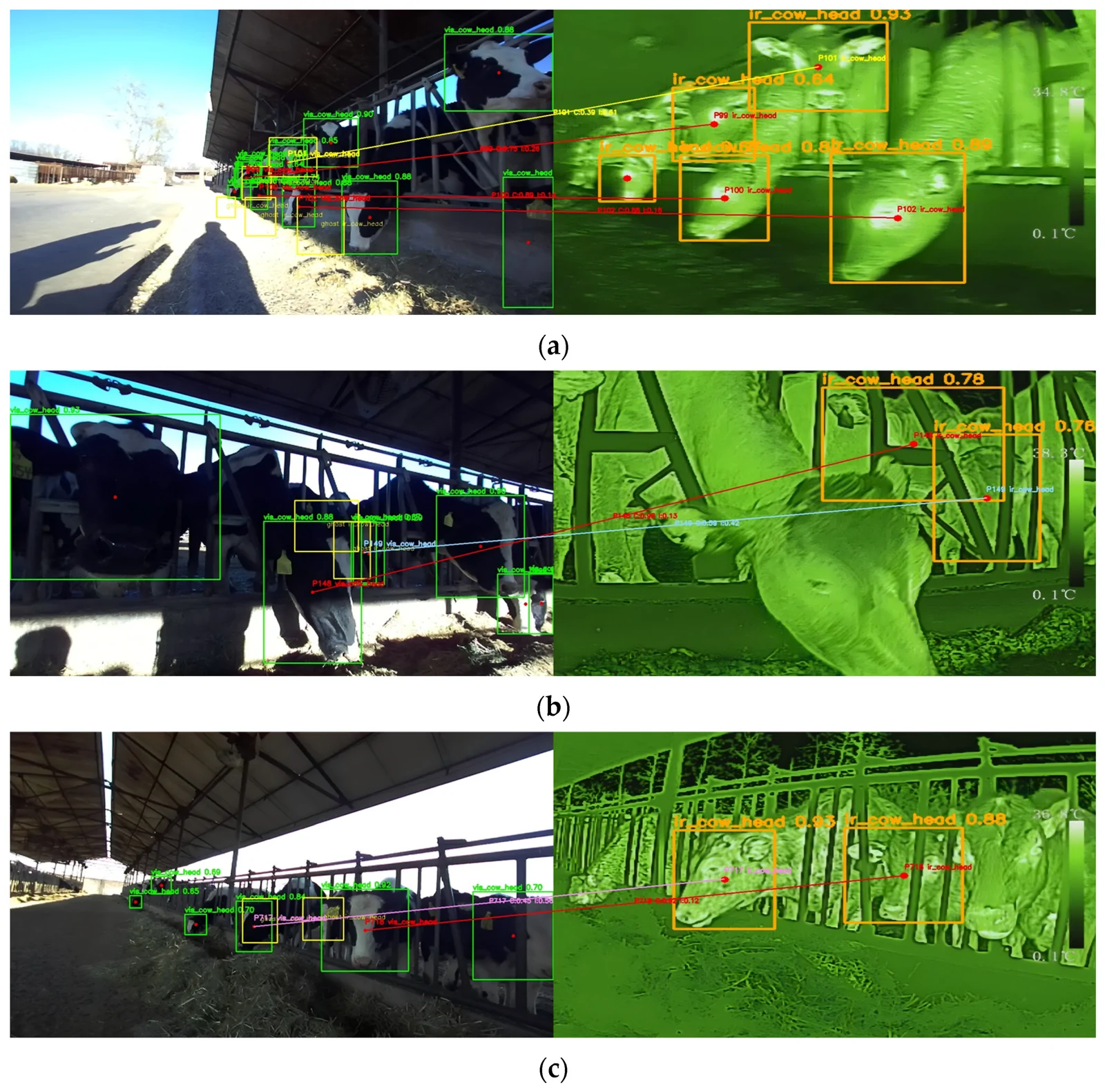
Representative heterogeneous-camera target-association failure cases. (**a**) Incorrect associations and an unassociated infrared target during robot turning in a five-cow scene under strong-light interference; (**b**) visible-light missed detection under shadow interference, dense cow distribution, and viewpoint variation; (**c**) modality-specific missed detections in the same synchronized image pair, where dense cow distribution, fence occlusion, and weak illumination caused a visible-light missed detection, while the temperature-scale overlay caused an infrared missed detection.

Representative association failures are shown in Figure 13. During robot turning in a five-cow scene, strong-light interference and a substantial change in camera viewpoint altered the relative spatial arrangement of the cow-head targets between the two modalities. Four cow heads were incorrectly associated, and one infrared cow-head target remained unassociated.

In another dense multi-cow scene, shadow interference and viewpoint variation caused a cow head to be missed in the visible-light image. The resulting difference in the detected target sets affected the subsequent one-to-one association. In a further synchronized image pair, dense cow distribution, fence occlusion, and weak illumination caused a cow head to be missed in the visible-light image, while interference from the temperature-scale overlay caused a cow head to be missed in the infrared pseudo-colour image. These modality-specific missed detections changed the candidate target sets available to the association algorithm and prevented the corresponding cow-head pairs from being established.

The correct-association examples included single- and multi-cow scenes, partial fence occlusion, unequal detected target numbers, and spatial offsets between the two camera views. The failure cases were concentrated in scenes involving rapid viewpoint variation, dense target distributions, illumination interference, and modality-specific missed detections.

### 3.4. Edge-Device Deployment Performance Analysis

**Figure 14:**
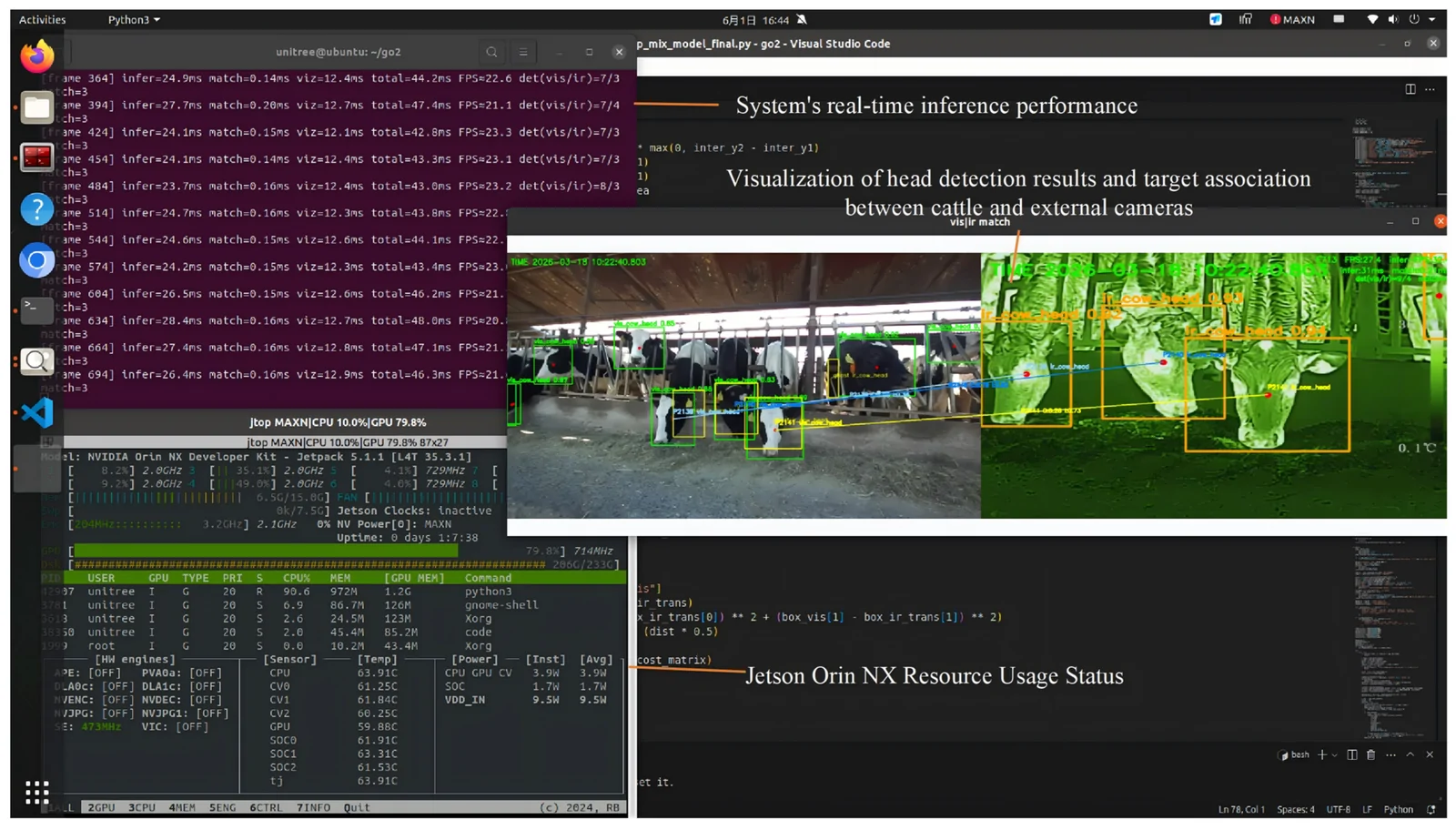
Deployment results on the inspection robot edge computing platform.

The proposed system was deployed on a quadruped inspection robot to evaluate real-time edge performance. The robot carried an NVIDIA Jetson Orin NX 16GB computing module, and the runtime environment included Jetpack 5.1.1, CUDA 11.4, cuDNN 8.6.0, and TensorRT 8.6.0. The platform provides about 100 TOPS of AI computing capability with a maximum power consumption of 25 W. Native PyTorch and TensorRT-accelerated inference were both tested. Figure 14 presents the deployment interface, and Table 5 summarizes average inference time, end-to-end processing time, and frame rate.

To evaluate the effects of model conversion and numerical precision, the same YOLO11-AFE checkpoint was tested using three inference configurations: PyTorch FP32, TensorRT FP32, and TensorRT FP16. All three configurations used the same labelled test dataset, an input resolution of 640 × 640 pixels, and identical evaluation settings. The modality-specific detection accuracy and average model inference time are reported in Table 5.

Before TensorRT conversion, the on-device PyTorch FP32 results were compared with the three-run mean values reported in Table 2. For the visible-light subset, precision, mAP@0.5, and mAP@0.5:0.95 differed by −0.08, −0.11, and −0.12 percentage points, respectively, whereas the corresponding differences for the infrared pseudo-colour subset were −0.18, −0.13, and −0.18 percentage points. Relative to the mean results in Table 2, the final TensorRT FP16 deployment reduced visible-light mAP@0.5 and mAP@0.5:0.95 by 1.923 and 1.517 percentage points, respectively, and reduced the corresponding infrared metrics by 1.223 and 2.550 percentage points.

As shown in Table 5, TensorRT substantially improved inference efficiency on the Jetson Orin NX. Compared with on-device PyTorch FP32 inference, TensorRT FP32 reduced the average inference time from 21.88 to 10.59 ms image^−1^, corresponding to a 51.60% reduction and an approximately 2.07-fold acceleration. TensorRT FP16 further reduced the inference time to 5.43 ms image^−1^, representing a 75.18% reduction and an approximately 4.03-fold acceleration relative to PyTorch FP32. Under TensorRT FP16 inference, visible-light precision, mAP@0.5, and mAP@0.5:0.95 decreased by 0.16, 1.81, and 1.40 percentage points, respectively, relative to the on-device PyTorch baseline, whereas the corresponding decreases for the infrared subset were 2.91, 1.09, and 2.37 percentage points. The larger reduction in infrared precision suggests that infrared detection was more sensitive to the TensorRT deployment pipeline.

The accuracy differences between TensorRT FP32 and TensorRT FP16 were limited. Switching from FP32 to FP16 changed the visible-light precision, mAP@0.5, and mAP@0.5:0.95 by 0.00, −0.09, and +0.01 percentage points, respectively, while the corresponding infrared changes were −0.08, +0.01, and +0.15 percentage points. Thus, all mAP differences between the two TensorRT precision modes were within 0.15 percentage points, whereas FP16 reduced inference time by a further 48.73%. These results indicate that FP16 introduced little additional accuracy variation after TensorRT conversion and provided the most favourable balance between detection performance and inference efficiency. The inference time in Table 5 was measured per individual image during model-accuracy evaluation, whereas Table 6 reports the time required to process one synchronized visible–infrared image pair within the complete deployed pipeline.

Under PyTorch FP32, the average inference time was 38.14 ms, giving 26.22 FPS. Including inference, bounding-box parsing, and target association, the core process required 40.81 ms, equivalent to 24.50 FPS. With TensorRT, inference time fell to 24.23 ms and the inference speed increased to 41.26 FPS; the core-process time was shortened to 27.24 ms or 36.71 FPS. Relative to PyTorch, TensorRT reduced inference time by 36.47%, raised inference FPS by 57.36%, reduced core-process time by 33.25%, and increased core-process FPS by 49.84%.

The target-association stage required an average of only 0.12 ms per synchronized image pair under both inference configurations. It accounted for 0.289% of the PyTorch core-process time and 0.444% of the TensorRT core-process time, indicating that target association was not the computational bottleneck of the pipeline. Overall, the TensorRT FP16 configuration provided the most favourable balance between detection accuracy and processing efficiency and supported real-time cow-head detection and heterogeneous-camera target association under the tested robotic inspection conditions.

## 4. Discussion

This study developed a compact cow-head detector and a heterogeneous-camera association method for robotic barn inspection. A visible–thermal acquisition and analysis platform was constructed on a quadruped robot with edge computing. In complex dairy-farm scenes, the platform achieved real-time cow-head detection and cross-camera association, providing target-level correspondence between visible-light and infrared pseudo-colour images. Recent studies have shown that facial thermal regions in cattle, including the eyes, muzzle, nostrils, ears, and horns, can be automatically located or analyzed for non-contact temperature monitoring [6,8]. Therefore, the proposed method can serve as a front-end perception module for future facial-temperature extraction in robotic dairy monitoring.

### 4.1. Innovation

Previous cattle-tracking methods have associated animals across successive frames using combinations of spatial location, colour, texture, and deep appearance features [16]. In contrast, the present method addresses instantaneous association between heterogeneous camera views, where visible-light appearance features cannot be assumed to remain consistent with infrared pseudo-colour observations. The association cost therefore uses modality-independent spatial cues derived from the detected cow-head boxes rather than temporal trajectories or cross-modal appearance similarity. These works are useful for farm perception, but a mobile inspection robot must deal with additional factors, including crowded cows, fence occlusion, viewpoint variation, overexposure, weak illumination, and low-texture infrared images. Compared with those studies, the present work moves from single-modal cow detection to visible–thermal cow-head detection and target-level cross-camera association on a moving robot. The obtained correspondence between visible-light and infrared pseudo-colour images provides the first step toward later local registration, facial-region recognition, and radiometric temperature extraction.

The comparative experiments were carried out with representative detection models under the same conditions. All models used the same visible–thermal cow-head dataset, data split, input resolution, training epochs, and hardware platform. The results show that YOLO11-AFE maintained high accuracy and stable detection performance under complex illumination, weak infrared texture, and multi-cow scenes, while reducing parameters and computational cost. This balance between accuracy, robustness, and lightweight computation is important for practical deployment on inspection-robot edge hardware.

The target-association design is distinct from image-level cross-modal registration, cross-view re-identification, and temporal multi-camera tracking. Instead of aligning complete visible-light and infrared images, determining persistent animal identities, or relying on temporal trajectories, the proposed method uses cow-head detection boxes from each synchronized image pair as the association units. The cost matrix integrates IoU and the relative centre-distance difference with respect to the corresponding image centres, after which the improved Hungarian association algorithm determines a global one-to-one assignment and rejects abnormal associations. Owing to this instantaneous target-level formulation, the method has low computational cost and is suitable for real-time processing on embedded robot hardware.

### 4.2. Application Scenario

The proposed method can serve as the front-end perception module for non-contact facial-temperature monitoring during robotic dairy inspection. It detects cow heads in synchronized visible-light and infrared pseudo-colour images and associates detections belonging to the same animal across the two camera views. This target-level correspondence is important in multi-cow scenes, because thermal information must be assigned to the correct individual before it can support animal-level monitoring.

**Figure 15:**
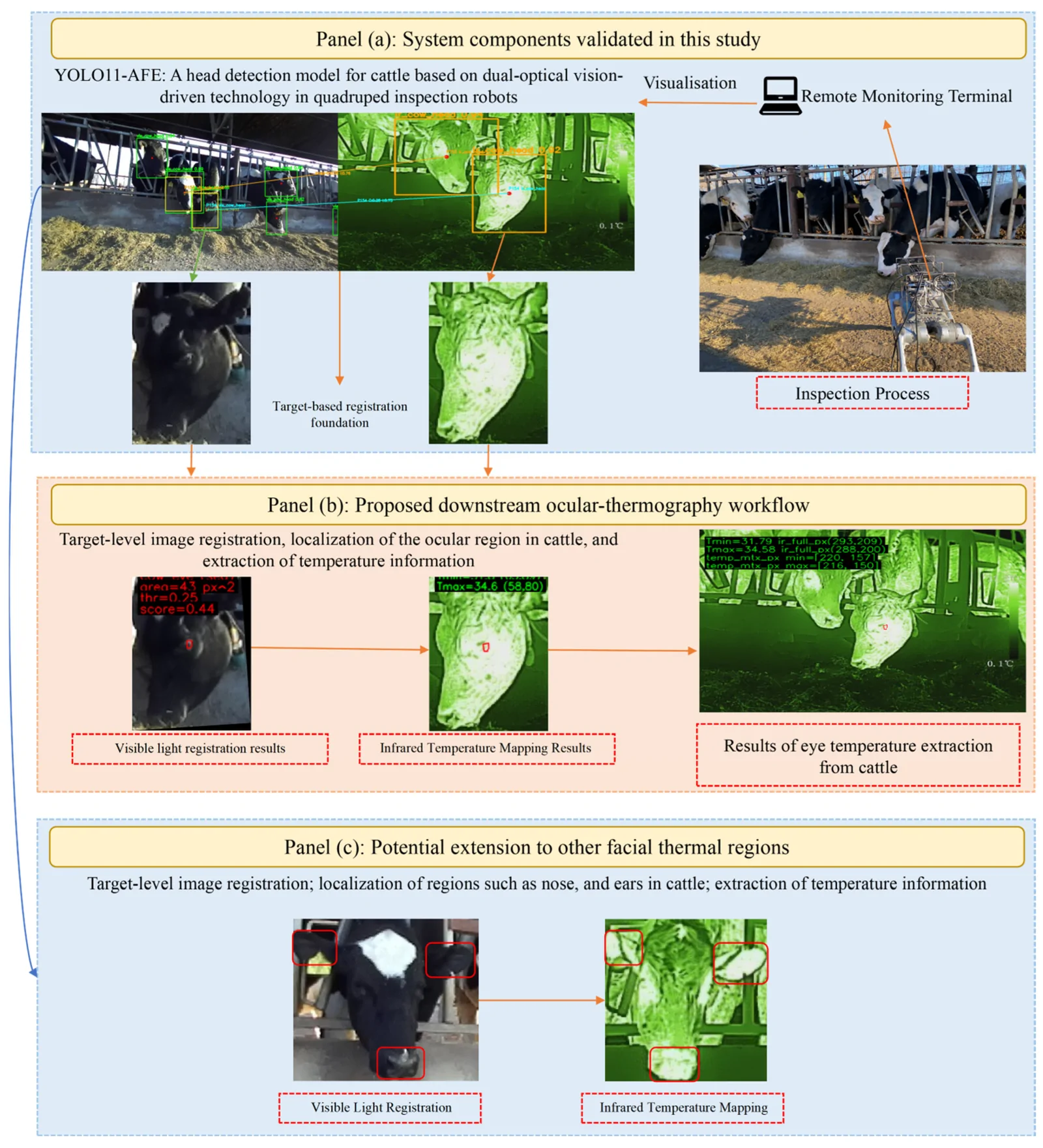
Workflow and application scenario of cow-head detection and heterogeneous-camera target association in the inspection robot vision system.

As shown in Figure 15, the potential workflow includes visible–thermal image acquisition, cow-head detection, heterogeneous-camera target association, local registration of matched head regions, facial-region recognition, and temperature extraction from the corresponding infrared temperature matrix. Visible-light images provide structural cues for stable localization, whereas infrared images provide thermal information. Therefore, the proposed method can provide a practical basis for future robotic monitoring of facial thermal regions such as the eyes, muzzle, nostrils, ears, and horns [5,6,8,30,31].

### 4.3. Limitations and Future Work

Several limitations remain. First, the present study verified cow-head detection, cross-camera association, and edge deployment, but it did not complete the full facial-temperature extraction pipeline. Future work should combine the proposed method with local visible–thermal registration, facial-region recognition, radiometric temperature extraction, and individual identification.

Second, the present system still depends partly on visible-light image quality. At night or under extremely weak illumination without auxiliary lighting, visible images may lose clear contours, texture details, and facial-region cues, which can affect head detection, target separation, and later visible-to-infrared local registration. Infrared pseudo-colour images are less affected by ambient visible light and can provide stable thermal radiation, but their limited texture is insufficient for independent fine localization of specific facial regions. Thus, the current system is best suited to daytime, indoor lighting, or scenarios with supplementary illumination. Future research should exploit the complementarity between visible structural information and infrared thermal information more deeply to improve night-time and low-light robustness [32].

Third, the current infrared pseudo-colour subset contains relatively few highly crowded scenes. Most infrared images contain one or three annotated cow heads, and scenes containing more than five clearly visible cow heads are insufficiently represented. This imbalance may limit the generalisability of the detector to high-density infrared scenes involving extensive target overlap and severe occlusion, and it should also be considered when comparing modality-specific detection results. Future work will expand the infrared dataset by collecting additional samples with higher cow densities, greater target overlap, more severe fence occlusion, and a wider range of robot-to-animal distances. The expanded dataset will be used to further evaluate detection robustness and cross-modal target association under more challenging crowded conditions [33].

## 5. Conclusions

This work investigated dual-light perception for inspection robots and focused on cow-head detection and cross-modal association in visible-light and infrared pseudo-colour images. A compact YOLO11-AFE detector and an improved Hungarian association algorithm were developed. The conclusions are as follows.

- By introducing ADown, C3k2_FE, and SPPF_ECA, YOLO11-AFE improved cow-head detection in both modalities. Compared with YOLO11n, visible-light precision, mAP@0.5, and mAP@0.5:0.95 increased by 4.743, 2.240, and 1.554 percentage points, respectively. The corresponding improvements on the infrared pseudo-colour subset were 1.846, 0.290, and 1.157 percentage points.
- The detector contained 2.14M parameters and required 5.27 GFLOPs, which are 17.37% and 18.17% lower than YOLO11n. Therefore, the model reduces computational demand while improving accuracy, making it appropriate for real-time cow-head detection on inspection-robot edge hardware.
- The improved Hungarian association algorithm uses both target-box IoU and the Euclidean distance difference relative to the image centre. Across the combined validation and test subsets, 364 of 371 manually confirmed cow-head pairs were correctly associated, six were incorrectly associated, and one remained unassociated, resulting in an overall association accuracy of 98.11%. These results indicate that the method can provide reliable cross-modal target-level correspondence for subsequent facial-region localization and radiometric temperature extraction.

## Figures and Tables

**Table 1:** Statistical distribution of the dataset (Unit: sheets).

Number of Cattle/Figure	Visible Images (VIS)			Infrared Pseudo-Colour Image (IR)		
	Train	Val	Test	Train	Val	Test
1	3	1	1	544	74	72
2	7	1	1	297	38	39
3	73	4	4	113	7	8
4	104	17	18	19	2	2
5	222	27	23	5	1	1
6	202	24	26	0	0	0
7	174	20	22	0	0	0
8	81	6	8	0	0	0
9	40	9	5	0	0	0
10	35	2	4	0	0	0
11–16	37	11	10	0	0	0
Total	978	122	122	978	122	122

**Table 2:** Detection results of different models on visible and infrared pseudo-colour thermal image test sets (mean ± standard deviation over three runs).

Model	Modality	P/%	mAP@0.5/%	mAP@0.5:0.95/%	Params(M)	FLOPs(G)
RT-DETR_L	VIS	92.733 ± 0.2157	93.007 ± 0.2082	67.793 ± 0.2146	32.81	108
	IR	89.223 ± 0.1701	95.973 ± 0.6117	88.457 ± 0.4215	32.81	108
Faster-RCNN	VIS	93.657 ± 0.4050	86.667 ± 0.4051	60.713 ± 0.6108	41.3	267.86
	IR	95.000 ± 0.3051	91.977 ± 0.2359	82.733 ± 0.5072	41.3	267.86
YOLOV5n	VIS	95.387 ± 0.1935	94.970 ± 0.6673	68.920 ± 0.3936	2.51	7.18
	IR	95.840 ± 0.0624	98.627 ± 0.1422	89.823 ± 0.5294	2.51	7.18
YOLOV8n	VIS	96.170 ± 0.2000	95.170 ± 0.3274	69.973 ± 0.4362	3.01	8.2
	IR	93.270 ± 0.2358	98.547 ± 0.3118	90.317 ± 0.1960	3.01	8.2
YOLOV10n	VIS	96.480 ± 0.3279	95.867 ± 0.1222	68.083 ± 0.3614	2.71	8.4
	IR	94.593 ± 0.3134	97.210 ± 0.2163	90.453 ± 0.1801	2.71	8.4
YOLO11n	VIS	92.847 ± 0.3496	94.133 ± 0.3855	69.203 ± 0.1504	2.59	6.44
	IR	94.267 ± 0.5150	98.783 ± 0.1457	90.343 ± 0.2747	2.59	6.44
YOLO12n	VIS	95.770 ± 1.7840	94.177 ± 0.2969	68.660 ± 0.4173	2.57	6.48
	IR	94.797 ± 0.2084	97.690 ± 0.2364	89.970 ± 0.2406	2.57	6.48
YOLO11-AFE	VIS	97.590 ± 0.2022	96.373 ± 0.2409	70.757 ± 0.5052	2.14	5.27
	IR	96.113 ± 0.2386	99.073 ± 0.0950	91.500 ± 0.4026	2.14	5.27

**Table 3:** Ablation experiment results (mean ± standard deviation over three runs).

ADown	C3k2_FE	SPPF_ECA	Modality	P/%	mAP@0.5/%	mAP@0.5:0.95/%	Params(M)	FLOPs(G)
-	-	-	VIS	92.847 ± 0.3496	94.133 ± 0.3855	69.203 ± 0.1504	2.59	6.44
			IR	94.267 ± 0.5150	98.783 ± 0.1457	90.343 ± 0.2747	2.59	6.44
√	-	-	VIS	97.187 ± 0.2021	94.737 ± 0.0551	69.630 ± 0.3305	2.04	5.17
			IR	95.770 ± 0.1562	98.803 ± 0.2710	91.317 ± 0.3153	2.04	5.17
-	√	-	VIS	95.893 ± 0.1804	95.997 ± 0.1193	70.860 ± 0.3617	2.62	6.46
			IR	96.030 ± 0.1513	98.890 ± 0.1609	91.477 ± 0.1124	2.62	6.46
-	-	√	VIS	96.477 ± 0.3007	95.737 ± 0.0751	69.550 ± 0.3593	2.59	6.44
			IR	94.143 ± 0.2996	98.940 ± 0.1735	91.163 ± 0.2003	2.59	6.44
√	√	-	VIS	96.773 ± 0.1222	95.017 ± 0.2491	69.163 ± 0.1604	2.14	5.27
			IR	96.867 ± 0.4302	98.963 ± 0.1305	90.987 ± 0.2060	2.14	5.27
√	-	√	VIS	95.307 ± 0.2043	94.503 ± 0.2205	68.937 ± 0.3556	2.11	5.25
			IR	93.880 ± 0.4952	98.583 ± 0.0321	90.400 ± 0.2066	2.11	5.25
-	√	√	VIS	97.497 ± 0.3153	95.643 ± 0.0945	70.470 ± 0.4703	2.62	6.46
			IR	93.893 ± 0.0643	98.757 ± 0.2403	91.323 ± 0.0643	2.62	6.46
√	√	√	VIS	97.590 ± 0.2022	96.373 ± 0.2409	70.757 ± 0.5052	2.14	5.27
			IR	96.113 ± 0.2386	99.073 ± 0.0950	91.500 ± 0.4026	2.14	5.27

Note:“√” indicates that the module is used, while “-“ indicates that the module is not used.

**Table 4:** Heterogeneous-camera target-association results on the validation and test subsets.

Evaluation Partition	Synchronized Image Pairs	Ground-Truth Cow-Head Pairs	Correct Associations	Incorrect Associations	Unassociated Pairs	Association Accuracy/%
Validation	122	184	177	6	1	96.20
Test	122	187	187	0	0	100.00
Combined evaluation	244	371	364	6	1	98.11

**Table 5:** Modality-specific detection accuracy and average inference time of YOLO11-AFE using PyTorch and TensorRT on Jetson Orin NX.

Inference Backend	Numerical Precision	VIS P/%	VIS mAP@0.5/%	VIS mAP@0.5:0.95/%	IR P/%	IR mAP@0.5/%	IR mAP@0.5:0.95/%	Inference Time/(ms Image^−1^)
PyTorch	FP32	97.51	96.26	70.64	95.93	98.94	91.32	21.88
TensorRT	FP32	97.35	94.54	69.23	93.10	97.84	88.80	10.59
TensorRT	FP16	97.35	94.45	69.24	93.02	97.85	88.95	5.43

**Table 6:** Runtime performance of the YOLO11-AFE system on Jetson Orin NX using PyTorch FP32 and TensorRT FP16 inference.

Inference Mode	Numerical Precision	Frame Reading Time/ms	Inference Time/ms	Bounding-Box Parsing Time/ms	Target-Association Time/ms	Core-Process Time/ms	Inference FPS	Core-Process FPS	Association Proportion/%
PyTorch	FP32	2.95	38.14	2.55	0.12	40.81	26.22	24.50	0.289
TensorRT	FP16	3.21	24.23	2.89	0.12	27.24	41.26	36.71	0.444

## Data Availability

The original contributions presented in this study are included in the article/Supplementary Materials. Further inquiries can be directed to the corresponding authors.

## Supplementary Materials

The following supporting information can be downloaded at: https://www.mdpi.com/article/10.3390/ani16162528/s1. Table S1: Sensitivity analysis of the SSIM threshold for temporal redundancy filtering; Table S2: Barn-level coverage of illumination and cow-density conditions before and after SSIM-based re-dundancy filtering; Table S3: Sensitivity of heterogeneous-camera target association to the post-assignment thresholds on the validation subset.

## Abbreviations

The following abbreviations are used in this manuscript:

ADown	Adaptive Downsampling
AFE	Adaptive Feature Enhancement
C3k2_FE	C3k2 with Feature-Adaptive Enhancement
ECA	Efficient Channel Attention
FiLM	Feature-wise Linear Modulation
FLOPs	Floating-Point Operations
FPS	Frames Per Second
GFLOPs	Giga Floating-Point Operations
IoU	Intersection over Union
IR	Infrared
IRT	Infrared Thermography
mAP	Mean Average Precision
NMS	Non-Maximum Suppression
RGB	Red–Green–Blue
SPPF	Spatial Pyramid Pooling-Fast
SPPF_ECA	Spatial Pyramid Pooling-Fast with Efficient Channel Attention
SSIM	Structural Similarity Index Measure
VIS	Visible-light Image
YOLO	You Only Look Once
