# Supplementary material for: An Edge-Deployable Method for Cow-Head Detection and Cross-Camera Association in Visible–Thermal Robotic Dairy Monitoring
Source: Animals (Basel). 2026 Aug 13;16(16):2528. doi: 10.3390/ani16162528 (PMC13508911; doi:10.3390/ani16162528)
Supplement: Supplementary file 1 [file animals-16-02528-s001.zip › animals-4468891-supplementary.pdf]

Supplementary Materials

Table S1. Sensitivity analysis of the SSIM threshold for temporal redundancy filtering.

SSIM Threshold	Raw Image Pairs	Retained Image Pairs	Removed Image Pairs	Retention Rate/%
0.65	6973	767	6206	11
0.7	6973	991	5982	14.21
**0.75**	**6973**	**1222**	**5751**	**17.52**
0.8	6973	1514	5459	21.71
0.85	6973	2317	4656	33.23
0.9	6973	3855	3118	55.28

Note: SSIM-based filtering was performed independently within each of the ten barn sequences. A temporally adjacent synchronized image pair was treated as redundant when its SSIM value was equal to or greater than the specified threshold. Consequently, lower thresholds resulted in more aggressive redundancy removal and lower retention rates.

Table S2. Barn-level coverage of illumination and cow-density conditions before and after SSIM-based redundancy filtering.

Predominant Barn Condition	Barns Before Filtering	Barns Represented After Filtering at 0.75	Dense-Cow Barns Before Filtering	Dense-Cow Barns Represented After Filtering at 0.75
Weak illumination	3	3	2	2
Strong-light interference	2	2	1	1
Normal natural illumination	2	2	2	2
Shadow-affected illumination	3	3	2	2
Total	10	10	7	7

Note: Each barn was assigned to one predominant illumination category according to the environmental conditions during data acquisition. Dense cow distribution was treated as an additional overlapping condition. SSIM-based redundancy filtering was performed independently within each barn sequence. All ten barns, including all seven barns characterized by dense cow distributions, remained represented after filtering at the selected threshold of 0.75.

Table S3. Sensitivity of heterogeneous-camera target association to the post-assignment thresholds on the validation subset.

Parameter Varied	T_C	T_d	T_{IoU}	Hungarian Assignments	Correct Associations	Incorrect or Unassociated Pairs	Correct Rate/%
**Selected setting**	**1.000**	**0.300**	**0.050**	**184**	**177**	**7**	**96.20%**
T_C	0.700	0.300	0.050	184	155	29	84.24%
T_C	0.750	0.300	0.050	184	162	22	88.04%
T_C	0.800	0.300	0.050	184	165	19	89.67%
T_C	0.850	0.300	0.050	184	168	16	91.30%
T_C	0.900	0.300	0.050	184	170	14	92.39%
T_C	0.950	0.300	0.050	184	171	13	92.93%
T_C	1.050	0.300	0.050	184	169	15	91.85%
T_C	1.100	0.300	0.050	184	165	19	89.67%
T_C	1.200	0.300	0.050	184	165	19	89.67%
T_d	1.000	0.050	0.050	184	136	48	73.91%
T_d	1.000	0.060	0.050	184	141	43	76.63%
T_d	1.000	0.080	0.050	184	156	28	84.78%
T_d	1.000	0.100	0.050	184	162	22	88.04%
T_d	1.000	0.150	0.050	184	165	19	89.67%
T_d	1.000	0.200	0.050	184	171	13	92.93%
T_d	1.000	0.250	0.050	184	173	11	94.02%
T_d	1.000	0.350	0.050	184	173	11	94.02%
T_d	1.000	0.400	0.050	184	170	14	92.39%
T_{IoU}	1.000	0.300	0.000	184	167	17	90.76%
T_{IoU}	1.000	0.300	0.025	184	173	11	94.02%
T_{IoU}	1.000	0.300	0.075	184	171	13	92.93%

T_{IoU}	1.000	0.300	0.100	184	170	14	92.39%
T_{IoU}	1.000	0.300	0.125	184	170	14	92.39%
T_{IoU}	1.000	0.300	0.150	184	169	15	91.85%
T_{IoU}	1.000	0.300	0.200	184	165	19	89.67%
T_{IoU}	1.000	0.300	0.250	184	165	19	89.67%
T_{IoU}	1.000	0.300	0.300	184	159	25	86.41%

Note: Threshold sensitivity was evaluated on the predefined validation subset containing 122 synchronized visible-light-infrared image pairs and 184 manually confirmed cow-head pairs. The selected threshold combination was T_C = 1.00, T_d = 0.30, and T_{IoU} = 0.05. During the local sensitivity analysis, one threshold was varied at a time while the other two were fixed at their selected values. The same detector outputs, geometric transformation parameters, distance-term weight, and ground-truth associations were used for all settings. Association accuracy was calculated as the number of correct associations divided by the 184 ground-truth association pairs.
